# The F-BAR protein pacsin2 inhibits asymmetric VE-cadherin internalization from tensile adherens junctions

**DOI:** 10.1038/ncomms12210

**Published:** 2016-07-15

**Authors:** Yvonne L. Dorland, Tsveta S. Malinova, Anne-Marieke D. van Stalborch, Adam G. Grieve, Daphne van Geemen, Nicolette S. Jansen, Bart-Jan de Kreuk, Kalim Nawaz, Jeroen Kole, Dirk Geerts, René J. P. Musters, Johan de Rooij, Peter L. Hordijk, Stephan Huveneers

**Affiliations:** 1 Department of Molecular Cell Biology, Sanquin Research and Landsteiner Laboratory, University of Amsterdam, Amsterdam 1066 CX, The Netherlands; 2 Department of Medical Biochemistry, Academic Medical Center, University of Amsterdam, Amsterdam 1105 AZ, The Netherlands; 3 Hubrecht Institute and University Medical Center Utrecht, Utrecht 3584 CT, The Netherlands; 4 Department of Medicine, University of California, San Diego, California 92093, USA; 5 Department of Physiology, VU University Medical Center, Amsterdam 1081 HV, The Netherlands; 6 Department of Pediatric Oncology/Hematology, Erasmus University Medical Center, Rotterdam 3015 GE, The Netherlands; 7 Department of Molecular Cancer Research, Center for Molecular Medicine, University Medical Center Utrecht, Utrecht 3584 CG, The Netherlands

## Abstract

Vascular homoeostasis, development and disease critically depend on the regulation of endothelial cell–cell junctions. Here we uncover a new role for the F-BAR protein pacsin2 in the control of VE-cadherin-based endothelial adhesion. Pacsin2 concentrates at focal adherens junctions (FAJs) that are experiencing unbalanced actomyosin-based pulling. FAJs move in response to differences in local cytoskeletal geometry and pacsin2 is recruited consistently to the trailing end of fast-moving FAJs via a mechanism that requires an intact F-BAR domain. Photoconversion, photobleaching, immunofluorescence and super-resolution microscopy reveal polarized dynamics, and organization of junctional proteins between the front of FAJs and their trailing ends. Interestingly, pacsin2 recruitment inhibits internalization of the VE-cadherin complex from FAJ trailing ends and is important for endothelial monolayer integrity. Together, these findings reveal a novel junction protective mechanism during polarized trafficking of VE-cadherin, which supports barrier maintenance within dynamic endothelial tissue.

## Abstract (web summary)

Cell-cell adhesion of endothelial tissue, mediated by the adhesion molecule VE-cadherin, is tightly regulated. Here the authors show that the F-BAR domain protein pacsin2 is recruited to the trailing end of mechanically unbalanced Focal Adherens Junctions, where it inhibits internalization of VE-cadherin and protects cell-cell adhesion.

## Graphical abstract

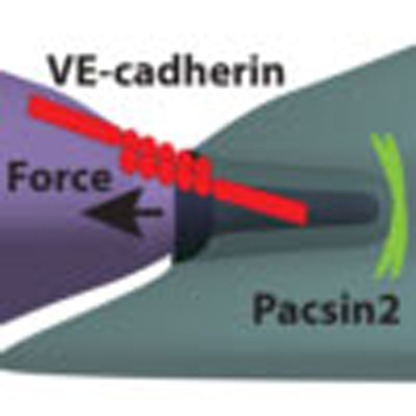

The endothelium is a cell monolayer that covers the luminal side of blood vessels and maintains vascular barrier function. Regulation of endothelial monolayer integrity in homoeostasis, as well as its remodelling during angiogenesis, and transendothelial trafficking of immune cells, occurs at the VE-cadherin adhesion complex, the central component of endothelial cell–cell junctions^1^,^2^. Perturbation of the tight balance between endothelial cell–cell junction stabilization and remodelling leads to developmental defects and vascular diseases, such as chronic inflammation, oedema and atherosclerosis^3^,^4^,^5^,^6^.

To keep barrier integrity in vascular homoeostasis, perturbations at endothelial cell–cell junctions, induced by the highly dynamic mechanical changes that blood vessels experience^7^, have to be compensated. Maintaining endothelial cell–cell adhesion relies on the capacity of junctions to adapt to changes in local forces they experience^8^. Interactions between cells in tissue are often collectively regulated and occur via polarized cell–cell junction dynamics^9^,^10^,^11^. For instance cells that lead processes of collective migration, such as during sprouting angiogenesis, generate actomyosin pulling forces and pull along follower cells^12^,^13^,^14^,^15^. Importantly, cadherin-based complexes are the adhesive entities that integrate these mechanical cues from leader cells to guide follower cells^16^. It is still unclear how endothelial cells maintain vascular integrity during local differences in actomyosin–mediated pulling.

The VE-cadherin complex, which comprises the core catenins that connect the transmembrane cadherins to the actin cytoskeleton^17^,^18^, is regulated by mechanical forces that are either external or induced by the actomyosin cytoskeleton^19^,^20^,^21^,^22^,^23^. We have previously shown that actomyosin pulling forces induce the formation of a distinct type of cell–cell junction: the focal adherens junction (FAJ)^24^. FAJs are connected to radial pulling F-actin bundles and contain the mechanotransduction protein vinculin. Force-dependent remodelling FAJs are prominent in monolayers of endothelial tissue cultures^24^, and in the endothelium of (remodelling) blood vessels^14^,^15^,^25^. Actomyosin-derived forces not only regulate the formation of FAJs and the interaction of the VE-cadherin complex with the actin cytoskeleton, but may also alter the plasma membrane by inducing local membrane curvatures^26^. Curved membranes are recognized and regulated by proteins that contain Bin–Amphiphysin–Rvs (BAR) domains^27^,^28^,^29^. In the current study we identify, using various imaging approaches, a completely novel asymmetric signalling event at adherens junctions. We uncover that unbalanced actomyosin activity near FAJs provides an asymmetric signal for recruitment of the Fer-CIP4 homology-BAR (F-BAR) protein pacsin2, an inhibitor of endocytosis^30^, in only one of the cells taking part in the junction. Polarized internalization of VE-cadherin occurs from the junctional side, where pacsin2 is recruited. We further demonstrate that pacsin2 recruitment is important for maintenance of cell–cell adhesion by stabilizing the VE-cadherin complex within FAJs and inhibition of its internalization.

## Results

### F-BAR protein recruitment to force-dependent junctions

**Figure 1:**
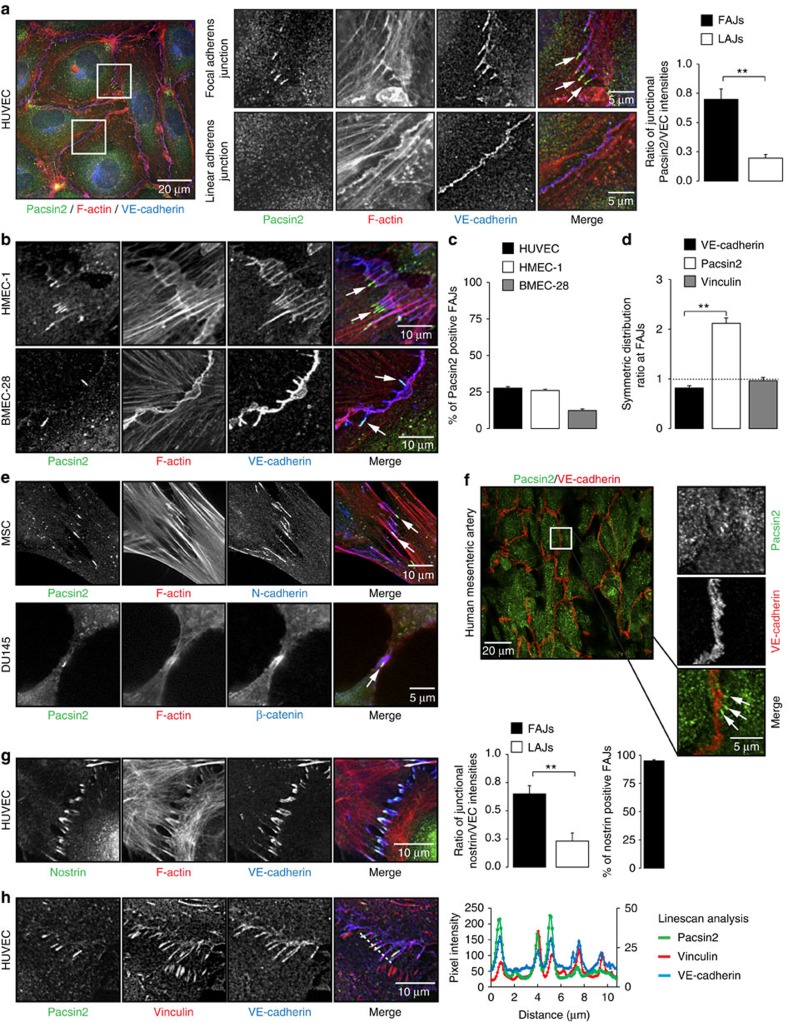
F-BAR protein recruitment to force-remodelling adherens junctions. (**a**) Widefield immunofluorescence (IF) images of HUVECs stained for pacsin2 (green), F-actin (red) and VE-cadherin (blue). Magnified images of regions of interest at focal adherens junctions (FAJs) and linear adherens junctions (LAJs). Arrows indicate pacsin2-positive FAJs. Graph shows the average ratio±s.e. of the mean (s.e.m.) between pacsin2 and VE-cadherin fluorescence integrated intensities along junctional linescans. *n*=44 FAJs; *n*=36 LAJs from three independent experiments. (**b**) Widefield IF images of FAJs in HMEC-1 and BMEC-28 monolayers stained for pacsin2 (green), F-actin (red) and VE-cadherin (blue). (**c**) Graph shows a quantification of the percentage±s.e.m. of FAJs that are pacsin2-positive in IF images of HUVEC (*n*=22 images), HMEC-1 (*n*=16 images) or BMEC-28 cells (*n*=30 images) from *n*≥2 independent experiments. (**d**) Graph shows the average distribution±s.e.m. of VE-cadherin, pacsin2 and vinculin intensities along pacsin2-positive FAJs. A ratio of 1 represents symmetric distribution along FAJs. For VE-cadherin and pacsin2, the analysis was from *n*=40 FAJs, and for vinculin *n*=27 FAJs from *n*≥2 independent experiments. (**e**) Widefield IF images of FAJs in mesenchymal stromal cells (MSC) stained for pacsin2 (green), F-actin (red) and N-cadherin (blue); and prostate epithelial cells (DU145) stained for pacsin2 (green), F-actin (red) and β-catenin (blue). (**f**) En face confocal IF images of human mesenteric artery stained for VE-cadherin (red) and pacsin2 (green). (**g**) Widefield IF images of FAJs in HUVEC monolayer stained for nostrin (green), F-actin (red) and VE-cadherin (blue). First graph shows the average ratio±s.e.m. between nostrin and VE-cadherin fluorescence integrated intensities along junctional linescans. *n*=33 FAJs; *n*=31 LAJs from three independent experiments. The second graph shows the average percentage±s.e.m. of FAJs that are nostrin positive (*n*=12 HUVEC images from three independent experiments). (**h**) Widefield IF images of FAJs in HUVECs stained for pacsin2 (green), vinculin (red) and VE-cadherin (blue). Linescan analysis along the white dotted line shows the fluorescence intensities of VE-cadherin (right *y* axis), vinculin and pacsin2 signals (left *y* axis). **P≤0.01 (Student's *t*-test).

The F-BAR protein pacsin2 (also known as syndapin2) is known to regulate the actin cytoskeleton^31^,^32^,^33^ and endocytosis^30^,^34^. While studying the role of pacsin2 in cytoskeletal remodelling during spreading of primary human umbilical vein endothelial cells (HUVECs)^33^, we observed that a subset of pacsin2 protein localizes near cell–cell junctions. This suggested that this F-BAR protein is involved in junction regulation. To investigate if pacsin2 associates with the VE-cadherin complex, we performed immunofluorescence (IF) on HUVEC monolayers and stained for endogenous pacsin2, VE-cadherin and F-actin. These IF stainings show that pacsin2 is specifically recruited to VE-cadherin-based FAJs, which appear as perpendicularly oriented junction fragments between cells^24^, but not to stable linear adherens junctions (Fig. 1a). IF stainings of pacsin2, VE-cadherin and F-actin in human dermal microvascular endothelial cells (HMEC-1 s) and human bone marrow endothelial cells (BMEC-28 (ref. 35) corroborate these findings (Fig. 1b), indicating that the association of pacsin2 with FAJs is conserved in endothelial cells from different tissue origins. In high-magnification IF images, we observed two intriguing aspects of the pacsin2–FAJ association: first, pacsin2 is present only at a subset of FAJs (Fig. 1c); and second, pacsin2 does not precisely colocalize with VE-cadherin, but concentrates asymmetrically at one side of FAJs (Fig. 1a,b,d; Supplementary Movie 1). Moreover, pacsin2 is recruited to FAJs of mesenchymal stromal and epithelial cells, respectively (Fig. 1e), extending this finding to other classical cadherin-based adhesions. To study if pacsin2 is recruited to endothelial junctions in human vasculature, we applied IF on vessels that were isolated from healthy mesentery sections from colon resections^25^. En face confocal microscopy shows that pacsin2 is recruited to endothelial adherens junctions in human arteries (Fig. 1f). Subsequently, we investigated which other pacsin isoforms might be expressed in endothelial cells. Western blot analysis shows that small amounts of pacsin3, but not of pacsin1, are present in endothelial cell lysates (Supplementary Fig. 1a). IF experiments further indicate that endogenous endothelial pacsin3 is not detectable at FAJs (Supplementary Fig. 1b), suggesting that pacsin2 is the dominant pacsin isoform present at endothelial junctions. To investigate if the junctional recruitment of pacsin2 is specific for this F-BAR protein, we next analysed the distribution of nostrin, another F-BAR protein expressed in the endothelium^36^, and found that just like pacsin2, nostrin is recruited specifically to FAJs (Fig. 1g). In contrast to the recruitment of pacsin2 to a subset of FAJs, nostrin is recruited to almost all FAJs in HUVECs (95.0%±2.5 s.e.m.; Fig. 1g).

FAJs are not only phenotypically distinct from stable linear adherens junctions, but are also distinguished by the presence of vinculin, which interacts with the VE-cadherin–α-catenin complex upon force-induced tension^23^,^24^. To investigate the association of pacsin2 and vinculin with VE-cadherin at FAJs, we performed triple IF stainings. These experiments clearly show that pacsin2 and vinculin are both specifically present at FAJs (Fig. 1h). However, we observed that pacsin2 was recruited to the VE-cadherin–vinculin complex only at a fraction of FAJs (Fig. 1c,h), whereas vinculin is present at all FAJs^24^. Moreover, vinculin distribution at FAJs compares to that of VE-cadherin, whereas pacsin2 is asymmetrically present at one side of FAJs (Fig. 1d). These data argue against a direct force-induced molecular interaction of pacsin2 with the VE-cadherin–α-catenin complex itself, but indicate that pacsin2 is recruited to FAJs via a different mechanism. Because the F-BAR protein nostrin is recruited to FAJs as well, we propose that specific membrane curvature at FAJs recruits pacsin2. Together, these data reveal that F-BAR proteins are recruited to force-dependent remodelling junctions in vascular endothelium, as well as in other cell types that form cell–cell adhesions via classical cadherins.

### Unbalanced actomyosin activity at FAJs recruits pacsin2

**Figure 2:**
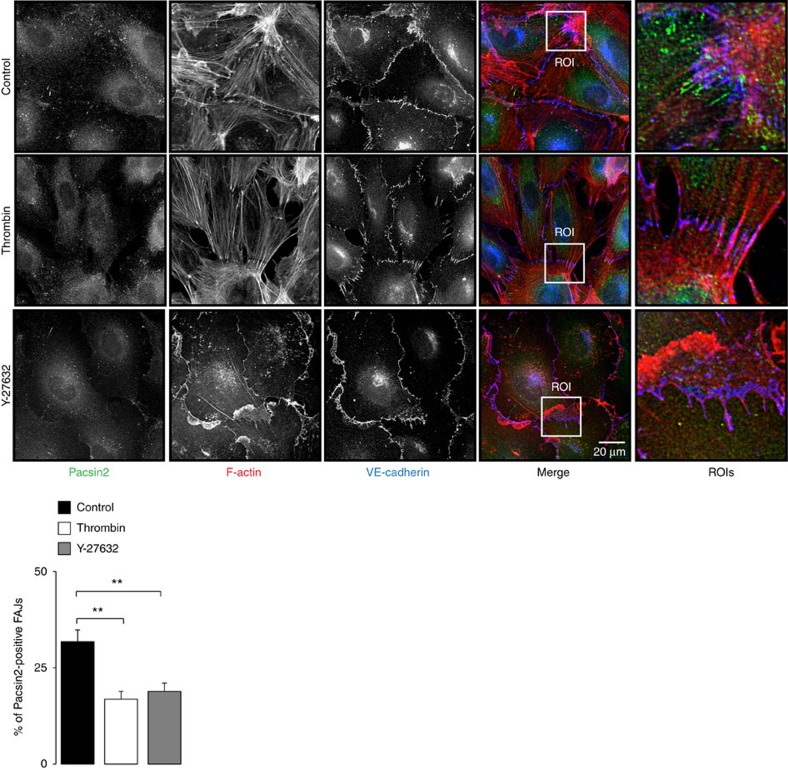
Regulation of actomyosin contractility and pacsin2 recruitment to FAJs. Widefield IF images of HUVECs treated with thrombin (0.2 U ml^−1^, 10 min) or the Rho-kinase inhibitor Y-27632 (10 μM, 10 min) as described previously^24^. Staining for pacsin2 is in green, F-actin in red and VE-cadherin in blue. The right panel represents enlargements of regions of interests from the merged images. Graph shows the average percentage±s.e.m. of FAJs that are pacsin2 positive from *n*≥2 independent experiments (control untreated HUVECs *n*=22 images; thrombin-treated HUVECs *n*=21 images; and Y-27632-treated HUVECs *n*=17 images). **P≤0.01 (Student's *t*-test).

**Figure 3:**
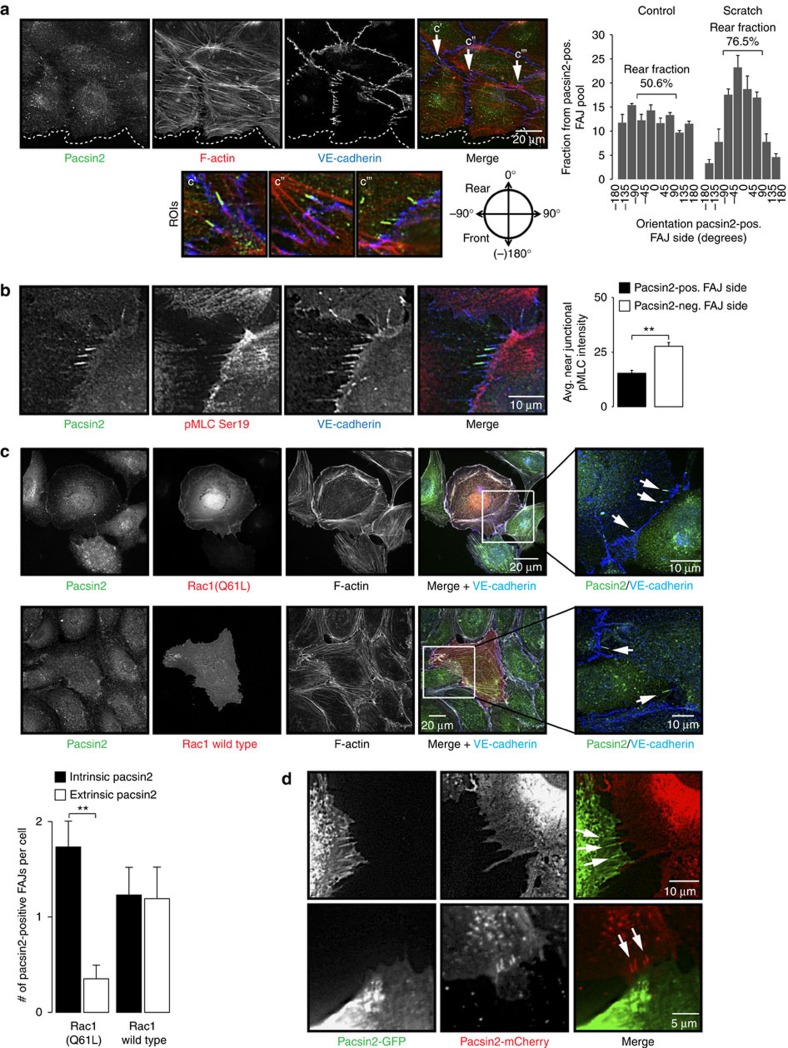
Asymmetric actomyosin organization between cells recruits pacsin2 to FAJs. (**a**) Widefield IF images of polarized migrating HUVECs in a scratch-wound assay that are stained for pacsin2 (green), F-actin (red) and VE-cadherin (blue). Arrows indicate the presence of pacsin2 at the rear ends of FAJs. Dotted line represents the leading front of the wound-closing HUVECs. The lower panels represent enlargements of three regions of interests from the merge image. Graph shows the average fraction from pacsin2-positive FAJs that are orientated in the indicated degree in relation to the scratch wound in IF images taken 4 h after wounding or in relation to the bottom (180°) of images of control monolayers. Analysis was performed on four independent experiments and included >250 junctions per experiment. (**b**) Confocal IF images of FAJs in HUVECs stained for pacsin2 (green), p-MLC Ser19 (red) and VE-cadherin (blue). Graph shows the average local integrated fluorescence intensity±s.e.m. of p-MLC Ser19 at the near junctional area at the pacsin2-positive versus pacsin2-negative side of FAJs. *n*=41 FAJs analysed from three independent experiments. (**c**) Widefield IF images of mosaic cultured HUVECs which are non-transfected or transfected with Rac1[Q61]-mCherry or wild-type Rac1-mCherry (red). Cells are stained for pacsin2 (green), F-actin (grey) and VE-cadherin (blue). Graph shows the average number of Pacsin2-positive FAJs in the transfected cells (intrinsic pacsin2) or in the neighbouring cells (extrinsic pacsin2). *n*=34 Rac[Q61L]-transfected cells and *n*=26 Rac1 wild-type-transfected cells from two independent experiments. Arrows indicate pacsin2-positive FAJs. (**d**) Widefield time-lapse images of mosaic FAJs between HUVECs with lentiviral expression of pacsin2-GFP (green) or pacsin2-mCherry (red). See also Supplementary Movie 4. **P≤0.01 (Student's *t*-test).

To understand how pacsin2 is recruited to cell–cell junctions, we first investigated the formation of FAJs by increasing cytoskeletal-derived tension on junctions, following thrombin treatments as described previously^24^. Strikingly, during the formation of FAJs by thrombin, we observed a decrease in the fraction of pacsin2-positive FAJs compared with control monolayers (Fig. 2; Supplementary Movie 2). Inhibition of cytoskeletal tension by using the Rho-kinase inhibitor Y-27632 also resulted in significantly fewer pacsin2-positive FAJs (Fig. 2; Supplementary Movie 3). These results suggest that the universal increases or decreases in tension on junctions in monolayers do not trigger pacsin2 recruitment. Next, we investigated pacsin2 recruitment to FAJs during polarized cell migration in scratch wound assays. IF stainings of control and scratch-wounded monolayers show that the polarized migration induces pacsin2 recruitment preferentially to the rear of FAJs compared with the direction of wound closure (Fig. 3a; the fraction of pacsin2-positive FAJs that recruited pacsin2 to the side opposite from the migration direction is 76.5%±3.95 s.e.m.). Subsequently, we investigated the level of actomyosin activity near pacsin2-positive FAJs. IF stainings for active myosin-II (myosin light chain phosphorylated on the Ser19 residue; pMLC-Ser19) showed that the near junctional distribution ratio of pMLC-Ser19 is unbalanced between cells sharing pacsin2-positive FAJs (Fig. 3b). The average ratio between pMLC-Ser19 intensity at the pacsin2-negative side and the intensity at the opposite side is 2.46±0.34 s.e.m. Thus, pacsin2 is recruited to the FAJ side opposite from the highest myosin-II-based pulling activity (Fig. 3b). These results indicate that differences in the geometry of the near junctional actomyosin network between cells relates to pacsin2 recruitment. The local organization of actomyosin at junctions is tightly controlled by the activation of small GTPases^37^,^38^,^39^. To study if forcing the actin cytoskeleton into an asymmetric geometry near FAJs directly regulates pacsin2 recruitment, we ectopically expressed an activated mutant of the GTPase Rac1 (Rac1[Q61L]) in HUVECs, and analysed their FAJs formed with untransfected cells in mosaic cultures. We find that pacsin2 is preferentially recruited to the FAJ side of cells expressing activated Rac1 (Fig. 3c), whereas pacsin2 recruitment in cells ectopically expressing wild-type Rac1 does not localize at a preferred FAJ side (Fig. 3c). Together, these data show that local asymmetric organization of the actomyosin cytoskeleton induces pacsin2 recruitment to FAJs.

We next addressed the mechanism that underlies the asymmetric presence of pacsin2 at junctions. A curve of the double membrane at AJs is intracellularly perceived both as convex and concave shapes, depending from which cellular side of the junction this is sensed. To investigate if the recruitment of pacsin2, known to interact with convex membranes^33^,^40^, to FAJs is derived from one or both cells forming the junction, we monitored dynamics of mosaic endothelial monolayers, in which half the cell population expresses lentivirally transduced pacsin2-green fluorescent protein (GFP) and the other half pacsin2-mCherry. These studies show that the presence of pacsin2 at FAJs is either GFP or mCherry fluorescent (Fig. 3d; Supplementary Movie 4), indicating that pacsin2 recruitment to junctions occurs from one side of the cell–cell junction.

**Figure 4:**
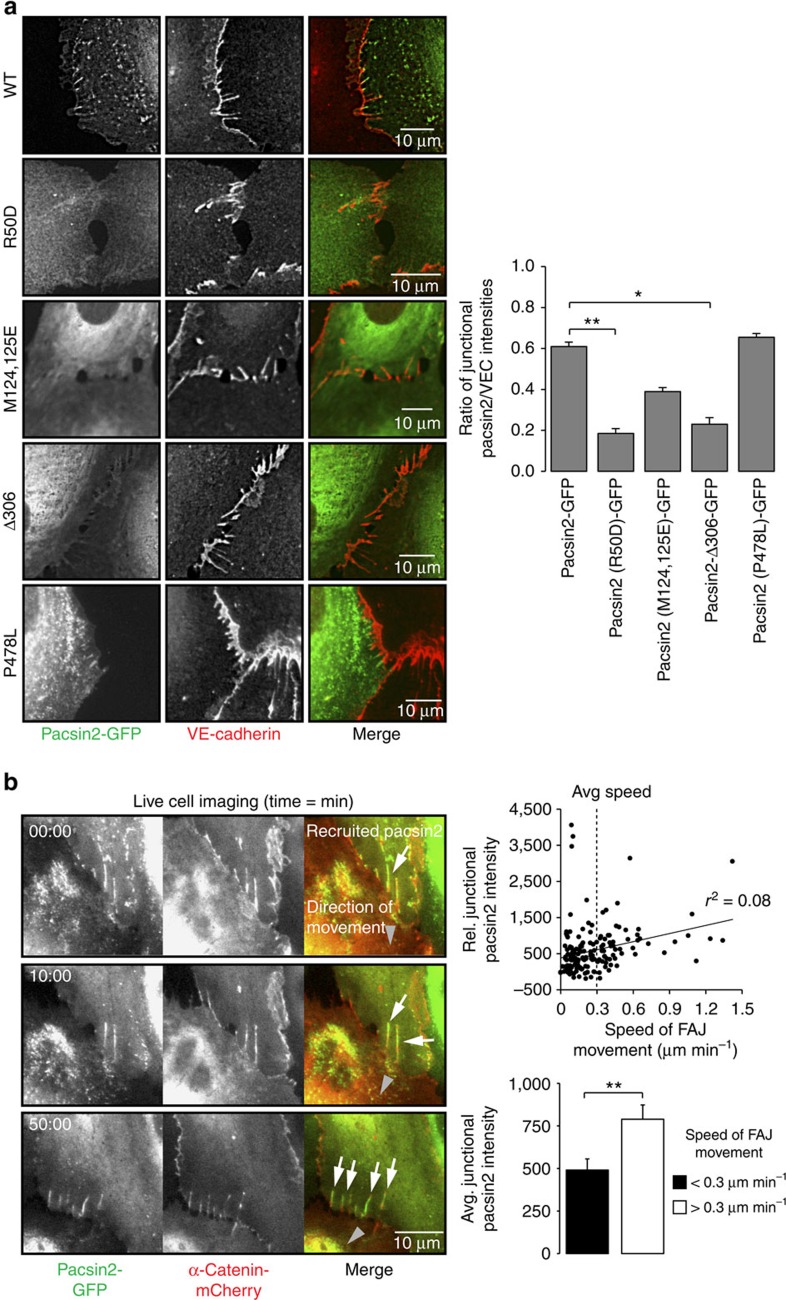
Pacsin2 is recruited to the trailing ends of fast-moving FAJs in an F-BAR domain-dependent manner. (**a**) Widefield IF images of HUVECs expressing indicated variants of pacsin2-GFP (green) that are stained for endogenous VE-cadherin (red). Graph shows the average ratio±s.e.m. between junctional pacsin2-GFP and VE-cadherin fluorescence integrated intensities from linescans along FAJs. Pacsin2, *n*=7 FAJs; pacsin2[R50D], *n*=17 FAJs; pacsin2[M124,125E], *n*=17 FAJs; pacsin2-Δ306, *n*=19 FAJs; and pacsin2[P478L], *n*=11 FAJs from two independent experiments. (**b**) Widefield time-lapse images of FAJs in HUVECs expressing pacsin2-GFP (green) and α-catenin-mCherry (red). White arrows indicate pacsin2 at junctions, grey arrowheads indicate the displacement direction of FAJs. Note that pacsin2 is recruited to the trailing ends of remodelling FAJs. See corresponding Supplementary Movie 5 for a ∼60 min time-lapse recording. See also Supplementary Movies 6, 7, 8. Scatter plot shows a quantification of the speed of FAJ movement in relation to the relative integrated fluorescence intensity of pacsin2-GFP from linescans along FAJs. The average speed of all analysed FAJs is 0.3 μm min^−1^. The bar graph is based on the same data set and shows the average junctional pacsin2 intensity at FAJs that move slower than 0.3 μm min^−1^ (*n*=109 FAJ tracks) or faster than 0.3 μm min^−1^ (*n*=56 FAJ tracks) from a total of nine independent time-lapse recordings. *P≤0.05; **P≤0.01 (Student's *t*-test).

Pacsin2, like many other F-BAR proteins, contains an F-BAR domain that is crucial for its binding to phospholipids in curved membranes and an SH3 domain, which interacts with effector proteins^27^. To further characterize its junctional recruitment mechanism, we expressed pacsin2 variants in HUVECs. We first tested if the pacsin2 F-BAR domain is involved in its asymmetric recruitment to FAJs. We expressed a GFP-tagged pacsin2-R50D mutant, which can no longer recognize convex membrane curvature, and a double mutant pacsin2[M124,125E], which cannot induce membrane tubulation^33^,^40^, in HUVECs. In particular the pacsin2[R50D] mutant is no longer concentrated at FAJs (Fig. 4a). Next, we expressed a truncated variant of pacsin2 (pacsin2-Δ306), which contains the complete F-BAR domain, but lacks Asn-Pro-Phe (NPF) motifs and the SH3 domain. Also pacsin2-Δ306 protein is significantly perturbed in its capacity to concentrate at FAJs. Finally, expression of an SH3-domain-mutated pacsin2[P478L] did not affect concentration of pacsin2 at FAJs (Fig. 4a). These results indicate that the pacsin2 F-BAR domain is crucial, but not sufficient, for its recruitment to FAJs.

To assess pacsin2 recruitment in relation to FAJ remodelling, we performed live imaging of HUVECs with lentiviral expression of pacsin2-GFP and α-catenin-mCherry (as marker for adherens junctions). Imaging of these transduced cells indicates that when pulling activity between cells is unbalanced, FAJs themselves displace in the direction of the cell that pulls away. This provides FAJs with a front (that is, the junction side of increased pulling) and a trailing end that follows the movement of the FAJ. Pacsin2 is consistently recruited to FAJ trailing ends (Fig. 4b; Supplementary Movies 4 and 5). We confirmed these findings in live cell imaging experiments of HUVECs expressing VE-cadherin-GFP and pacsin2-mCherry, or pacsin2-GFP and p120-catenin-mCherry (Supplementary Movies 6 and 7). These experiments also show that pacsin2 recruitment is highly dynamic; its presence at junctions is not constitutive, but relates to the movement of FAJs during unbalanced remodelling. We find that the increased speed of FAJ movement significantly correlates with a higher concentration of pacsin2 at FAJs (Fig. 4b). Moreover, live imaging of cells expressing pacsin2[R50D]-GFP and α-catenin-mCherry confirms that a functional F-BAR domain is crucial for the recruitment of pacsin2 to fast-moving FAJs (Supplementary Movie 8).

Taken together, these data show that asymmetric remodelling of FAJs, mediated by local actomyosin changes, induces pacsin2 recruitment to the trailing ends of FAJs in an F-BAR domain-dependent manner.

### Association of pacsin2 with VE-cadherin at super-resolution

**Figure 5:**
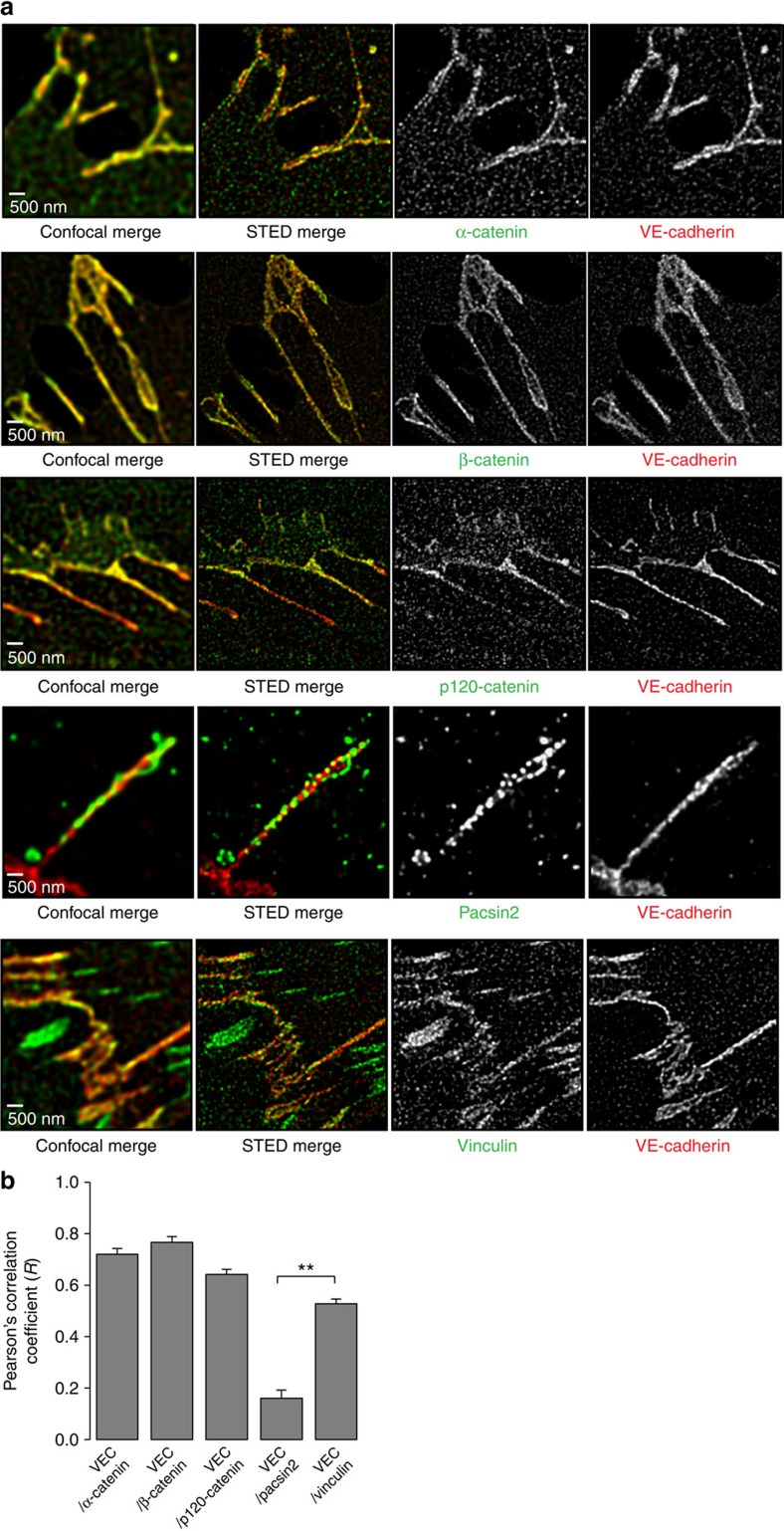
Low correlation of colocalization of pacsin2 and the VE-cadherin complex at super-resolution level. (**a**) Representative deconvolved images of stimulated emission depletion (STED) and confocal microscopy performed on IF labelled HUVECs stained for VE-cadherin (red) and catenins, vinculin or pacsin2 (all green). (**b**) Pearson correlation analysis of pixel intensities between VE-cadherin and indicated proteins at FAJ sections. Graph shows the average Pearson correlation coefficient (*R*)±s.e.m. of indicated protein pairs, which was determined on junctional region of interests (ROIs) from deconvolved STED images. *n*≥17 FAJs per correlation analysis. An *R* of 1 indicates perfect colocalization between two proteins, whereas an *R* of 0 indicates no correlation. **P≤0.01 (Student's *t*-test).

Because (i) recruitment of pacsin2 to FAJs occurs via a different mechanism than recruitment of vinculin, and (ii) pacsin2 recruitment depends on a functional F-BAR domain, which is known to interact with the plasma membrane, we next used stimulated emission depletion (STED) super-resolution microscopy to further dissect the molecular organization of FAJs. First, we analysed the colocalization of endogenous VE-cadherin with the core catenin proteins in STED images and, as expected, found a high Pearson's correlation coefficient (*R*) of these proteins with VE-cadherin at FAJs: α-catenin (*R*=0.72), β-catenin (*R*=0.77) and p120-catenin (*R*=0.64; Fig. 5a,b). Localization of vinculin also correlates well with that of VE-cadherin at this resolution (*R*=0.53). By contrast, pacsin2 molecules show a significantly lower correlation with VE-cadherin (*R*=0.16) and the super-resolution imaging reveals that pacsin2 is decorating the invaginating ends of FAJs (Fig. 5a,b). This detailed structure is not discernible in lower optic resolution imaging such as by conventional widefield (for example, Fig. 1a) or confocal imaging (Fig. 5a). Thus, the super-resolution imaging revealed that pacsin2 is not part of the VE-cadherin complex, but decorates around the VE-cadherin-based adhesion structure instead, suggesting this may be the location of curved membranes at asymmetric remodelling FAJs.

### Polarized dynamics of VE-cadherin at asymmetric FAJs

**Figure 6:**
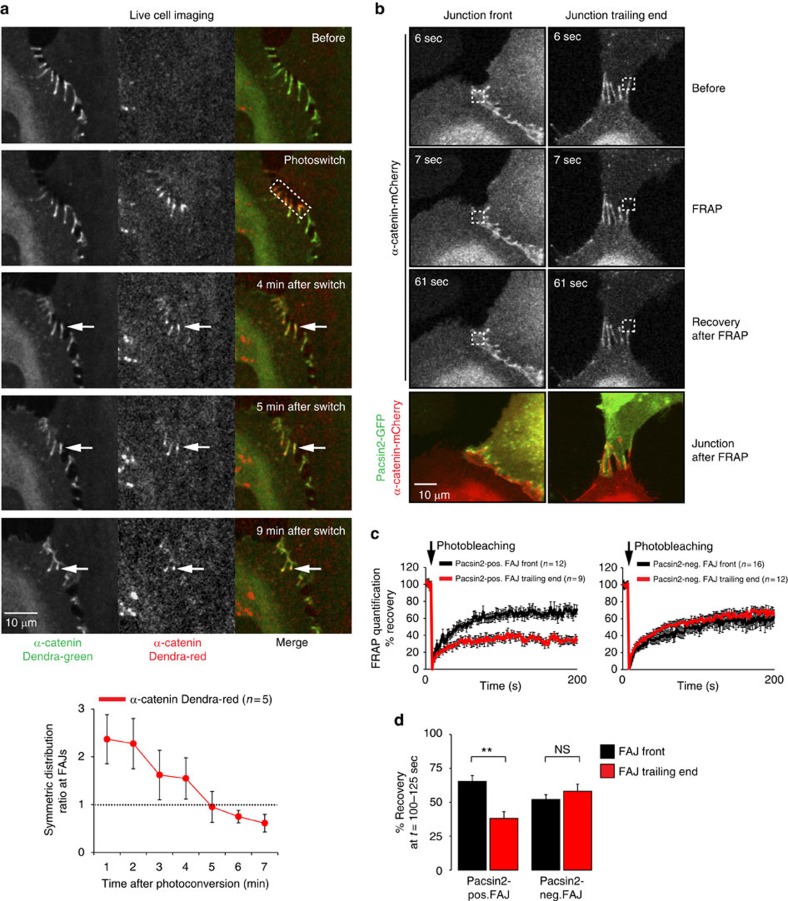
Polarized protein dynamics within asymmetric FAJs. (**a**) Time-lapse images of HUVECs expressing α-catenin-Dendra2 before and after photoswitching at the front of an FAJ using a 405-nm confocal laser. Newly recruited α-catenin protein (green) appears at the front of FAJs, whereas α-catenin-Dendra2 photoswitched to red moves to the trailing end of the FAJ (followed by the arrows). See corresponding Supplementary Movie 9 for a ∼12 min time-lapse recording. Line graph shows the average distribution±s.e.m. of photoconverted α-catenin-Dendra-red along FAJs in time. Symmetric ratio analysis was performed by splitting the integrated fluorescence intensities along linescans that span FAJs into two halves. A ratio >1 means concentration of α-catenin-Dendra-red at the FAJ front, a ratio of 1 means symmetric distribution along the FAJ, and <1 means concentration at the FAJ trailing end. *n*=5 independent Dendra photoswitching experiments. (**b**) Time-lapse images of HUVECs expressing pacsin2-GFP and α-catenin-mCherry showing mCherry fluorescence signal before and after photobleaching using a 561-nm confocal-laser to investigate protein dynamics at the front and at the trailing end of FAJs. Merged images at the bottom show pacsin2-GFP and α-catenin-mCherry localization of FAJs after the FRAP experiment. See corresponding Supplementary Movie 10 for a ∼200 s time-lapse recording. (**c**) Left graph shows the average recovery±s.e.m. of α-catenin-mCherry fluorescence signal as a percentage of the signal before bleaching at the front (12 independent FRAP experiments) or trailing end of pacsin2-positive FAJs (9 independent FRAP experiments). Right graph shows the quantification of similar FRAP experiments at pacsin2-negative FAJs (front, 16 independent experiments; trailing end, 12 independent experiments). (**d**) Bar graph shows the average±s.e.m. recovered mobile fraction of α-catenin-mCherry fluorescence signal at the front and trailing ends of pacsin2-positive and pacsin2-negative FAJs (based on the data of **c**). For pacsin2-negative FAJs, the junctional orientation was determined by the direction of observed junctional movement (*n*=16 independent experiments per junction side). The percentage of recovery was determined by calculating the average recovery in the plateau phase of the FRAP curves between 100 and 125 s after bleaching. **P-value≤0.01, NS, not significant (Student's *t*-test).

The previous experiments suggest the existence of a mechanism for polarized regulation of AJs in response to unbalanced cytoskeletal pulling between cells. The recruitment of pacsin2 associates with this polarization. To understand the consequences of asymmetric regulation of VE-cadherin-based junctions, we first expressed and visualized a fusion of α-catenin with the Dendra2 green-to-red photoswitchable fluorescent protein. Next, we photoswitched α-catenin-Dendra2 at the front of asymmetric FAJs. These experiments show that α-catenin molecules photoconverted to Dendra2-red at the front of FAJs move to the trailing ends of FAJs over time (Fig. 6a; Supplementary Movie 9). To investigate if there is differential mobility of junctional proteins in distinct parts of FAJs, we next performed fluorescence-recovery-after-photobleaching (FRAP) experiments of α-catenin-mCherry at pacsin2-positive FAJs. We compared α-catenin recovery at the front of pacsin2-positive FAJs, to its recovery at the trailing ends. Whereas fluorescence intensity of α-catenin-mCherry at the front of junctions readily recovers after bleaching, we find a marked reduction in the recovery of α-catenin at the side of FAJs that recruited pacsin2 (Fig. 6b,c; Supplementary Movie 10). To quantify these results, we determined the average fluorescence intensity values (corrected for overall bleaching and normalized to fluorescence intensity before (100%) and after (0%) bleaching) of multiple FRAP experiments. These data indicate that the mean mobile fraction of α-catenin-mCherry at the front of pacsin2-positive FAJs (65.5%±4.2 s.e.m.) is significantly higher than at their trailing ends (38.1%± 4.9 s.e.m.; Fig. 6d). In contrast, the average fluorescence recovery of α-catenin-mCherry at the front and trailing ends of pacsin2-negative FAJs is comparable (Fig. 6c,d). These data reveal a correlation between pacsin2 recruitment and local stabilization of α-catenin at the trailing ends of polarized FAJs. This is consistent with an inhibitory role of pacsin2 in endocytosis^30^. We further conclude that pacsin2 demarcates the junction side that is lacking nascent formation of VE-cadherin-based adhesions.

### Pacsin2 and asymmetric internalization of VE-cadherin

**Figure 7:**
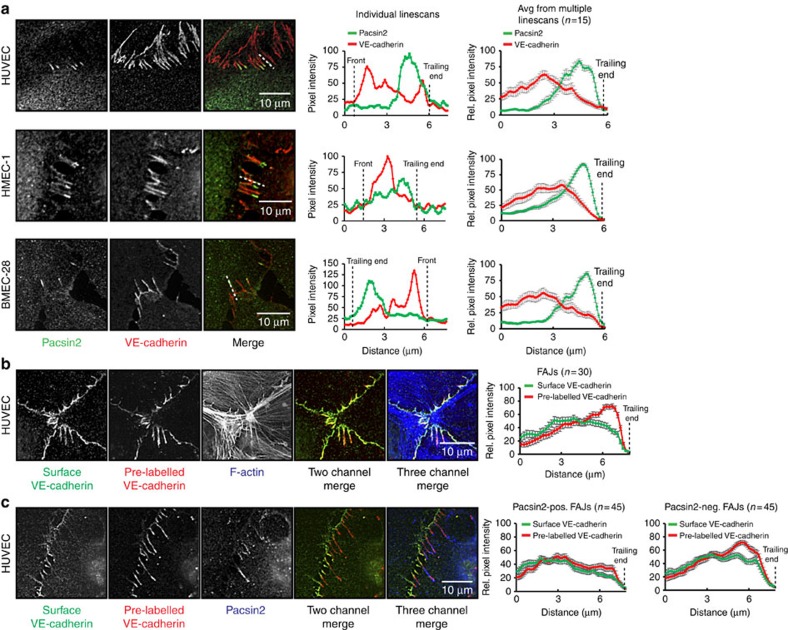
Pacsin2 associates with internalization of the VE-cadherin complex from FAJs. (**a**) Widefield IF images of FAJs in HUVEC, HMEC-1 and BMEC-28 cells stained for pacsin2 (green), and VE-cadherin (red). Front and trailing ends of FAJs are marked by black dotted lines in the individual linescan analysis. The right graphs display averages±s.e.m. of background-corrected and normalized VE-cadherin and pacsin2 intensities derived from multiple linescans along pacsin2-positive FAJs. (**b**,**c**) Widefield IF images of FAJs in HUVECs pre-labelled with an Alexa-Fluor-647-conjugated non-blocking anti-extracellular VE-cadherin antibody (mouse monoclonal 55/7H1). After labelling cells were cultured for 2 h, fixed and IF stained for surface VE-cadherin with mouse monoclonal 75 antibody. Note that the ‘old' surface VE-cadherin pool is internalized at the ends of FAJs (red colour in the merge), whereas new surface VE-cadherin molecules locate at the front of FAJs (green). Yellow indicates that VE-cadherin molecules were on the surface both at the moment of fixation and 2 h before fixation. Images of intracellular F-actin (**b**) or pacsin2 (**c**) (blue) were performed after permeabilizing the VE-cadherin-stained HUVECs. Graphs display averages±s.e.m. of background-corrected and normalized VE-cadherin intensities derived from multiple linescans along pacsin2-positive or pacsin2-negative FAJs.

**Figure 8:**
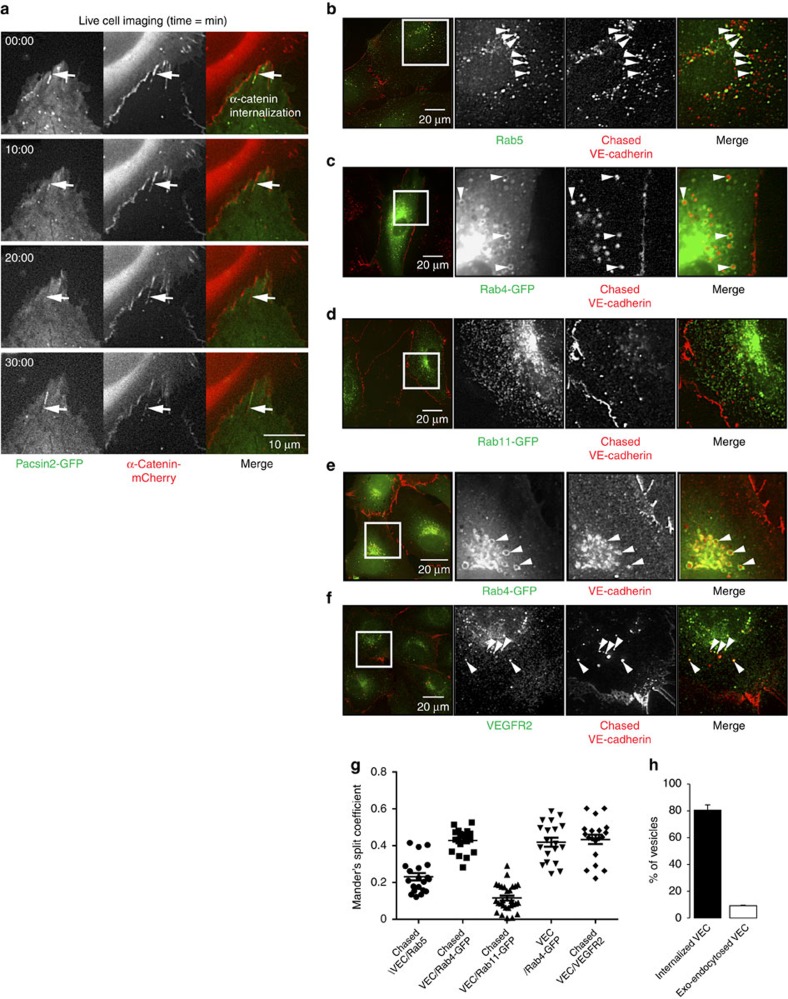
Pacsin2 association precedes VE-cadherin internalization. (**a**) Widefield time-lapse images of FAJs in HUVECs expressing pacsin2-GFP (green) and α-catenin-mCherry (red). Arrows follow α-catenin-mCherry internalizing from the junction to vesicles (red dots) and show pacsin2-GFP association during junctional internalization. See corresponding Supplementary Movie 11 for a 12-min time-lapse recording. (**b**) Widefield IF images of HUVECs pre-labelled with monoclonal 55/7H1 anti-VE-cadherin antibody and subsequently stained for Rab5. Arrowheads point to internalized VE-cadherin at Rab5-positive vesicles. (**c**,**d**) Widefield IF images of HUVECs expressing Rab4-GFP (**c**) or Rab11 (**d**) and live-labelled with monoclonal 55/7H1 anti-VE-cadherin antibody. Arrowheads point to internalized VE-cadherin at Rab4-positive vesicles. (**e**) Widefield IF images of HUVECs expressing Rab4-GFP and IF stained for total VE-cadherin. Arrowheads point to VE-cadherin at Rab4-positive vesicles. (**f**) Widefield IF images of HUVECs pre-labelled with monoclonal 55/7H1 anti-VE-cadherin antibody and subsequently stained for VEGFR2. Arrowheads point to internalized VE-cadherin at VEGFR2-positive vesicles. (**g**) Whisker dot plot shows the average±s.e.m. Mander's split coefficient (colocalization analysis) of pixel intensities of cytoplasmic VE-cadherin against indicated vesicular proteins. *n*≥19 cells per correlation analysis from *n*≥2 independent experiments. The Mander's split coefficient value indicates the fraction of detected VE-cadherin pixels colocalizing with the analysed vesicle proteins. (**h**) Graph shows the average portion of vesicles±s.e.m. that are positive for internalized VE-cadherin (black bar) or exo-endocytosed VE-cadherin (white bar). VE-cadherin-GFP was expressed in a sparse fraction of endothelial cells (donor cells) and VE-cadherin internalization was monitored in neighbouring adhered non-transduced cells (acceptor cells). Subsequently, we surface-chased VE-cadherin for 2 h using Alexa-647-labelled 55/7H1 antibody and determined the number of GFP+Alexa647+ (exo-endocytosed) versus GFP-Alexa647+ (internalized) vesicles in acceptor cells. Analysis was performed on *n*=27 cells from three independent experiments.

To understand the dynamics of VE-cadherin at the trailing ends of FAJs, we further investigated endogenous VE-cadherin and pacsin2 proteins in HUVEC, HMEC-1 and BMEC-28 monolayers. Since there is differential mobility of α-catenin, a crucial element of the VE-cadherin complex, between the front and trailing ends of pacsin2-positive FAJs, and because pacsin2 is known to inhibit internalization from the plasma membrane^30^, we next analysed the fluorescence intensities of VE-cadherin and pacsin2 across individual FAJs. Linescan analysis demonstrates that pacsin2 recruitment at the trailing ends of FAJs marks the junctional border, where the level of VE-cadherin protein declines (Fig. 7a), corresponding with their asymmetric difference in distribution along FAJs (Fig. 1d). To assess whether FAJ trailing ends are sites where VE-cadherin is being internalized, we live-labelled HUVEC monolayers with an Alexa-Fluor-647-conjugated non-blocking anti-extracellular VE-cadherin antibody (mouse monoclonal 55/7H1 (ref. 41). Two hours after the labelling, cells were fixed and stained for the surface VE-cadherin pool with another anti-extracellular VE-cadherin antibody (monoclonal 75; distinct fluorescence wavelength). IF images of these cells show that the pre-labelled VE-cadherin pool (representing those molecules that were on the surface 2 h before fixation) is concentrated at FAJ trailing ends (Fig. 7b). Under these conditions, the staining with clone 75 antibody was higher at FAJ fronts, suggesting that a significant portion of the VE-cadherin molecules is internalized from the cell surface at FAJ trailing ends (Fig. 7b). To investigate if pacsin2 recruitment correlates to the side of FAJs, where the majority of pre-labelled VE-cadherin molecules locate, we performed IF experiments in which pre-labelled surface VE-cadherin stainings were followed by permeabilization and stainings for pacsin2. These images show higher intensities of pre-labelled VE-cadherin than the surface pool of VE-cadherin at the pacsin2-positive trailing ends of FAJs (Fig. 7c). Of note, we noticed even more enrichment of pre-labelled VE-cadherin molecules at the trailing ends of pacsin2-negative FAJs (Fig. 7c). This is consistent with a stabilizing effect of pacsin2 on the mobility of α−catenin at the trailing ends of FAJs (Fig. 6d). Live imaging of HUVECs expressing pacsin2-GFP and α-catenin-mCherry further reveals that in some cases pacsin2 is recruited before internalization events from the trailing ends of FAJs. Internalization from those FAJs is promoted once pacsin2 dissociates from the junction (Fig. 8a; Supplementary Movie 11). Taken together these data indicate that pacsin2 is recruited to the internalization side of FAJs and that the presence of pacsin2 at FAJs correlates with suppressed levels of internalized VE-cadherin compared with pacsin2-negative FAJs.

To investigate the subcellular compartments to which internalized VE-cadherin is trafficked, we analysed the localization of surface-chased VE-cadherin molecules. We find that surface-chased VE-cadherin molecules culminate in Rab5 GTPase-positive endosomes (Fig. 8b,g), in line with other recent studies which show that endocytosis directs VE-cadherin to early endosomes^42^,^43^. Moreover, we find that a fraction (43%±2 s.e.m.) of surface-chased VE-cadherin is contained in Rab4-positive endosomes (Fig. 8c,g), but not in Rab11 recycling endosomes (Fig. 8d,g). The presence of VE-cadherin in Rab4-positive vesicles was confirmed in IF stainings for total endogenous VE-cadherin (Fig. 8e,g). Moreover, live cell imaging of VE-cadherin-red fluorescent protein (RFP) and Rab4-GFP shows that internalized VE-cadherin molecules that derive from the trailing ends of FAJs are directed towards Rab4-positive vesicles (Supplementary Movie 12). Colocalization analysis of surface-chased VE-cadherin molecules with the vascular endothelial growth factor receptor 2, known as cargo of recycling endothelial vesicles^44^, confirmed that VE-cadherin is targeted to an endosomal recycling compartment (Fig. 8f,g).

We next investigated what portion of internalized VE-cadherin molecules is intrinsically endocytosed, or alternatively, might be exo-endocytosed from neighbouring cells^43^,^45^. VE-cadherin-GFP was expressed in a sparse fraction of endothelial cells (donor cells) and VE-cadherin internalization in neighbouring adhered non-transduced cells (acceptor cells) was monitored. Subsequently, we surface-chased VE-cadherin for 2 h using Alexa-647-labelled 55/7H1 antibody and determined the number of GFP^+^/Alexa647^+^ versus GFP^-^/Alexa647^+^ vesicles in acceptor cells. These experiments show that 81% (±11.3 s.e.m.) of the internalized VE-cadherin-positive vesicles are endocytosed, whereas 9% (±1.4 s.e.m.) of the internalized vesicles were transferred from adjacent VE-cadherin expressing cells (Fig. 8h). Taken together, these data demonstrate that the VE-cadherin complex is internalized from asymmetric remodelling FAJs and transported to endosomal compartments.

### Pacsin2 controls VE-cadherin and endothelial barrier

**Figure 9:**
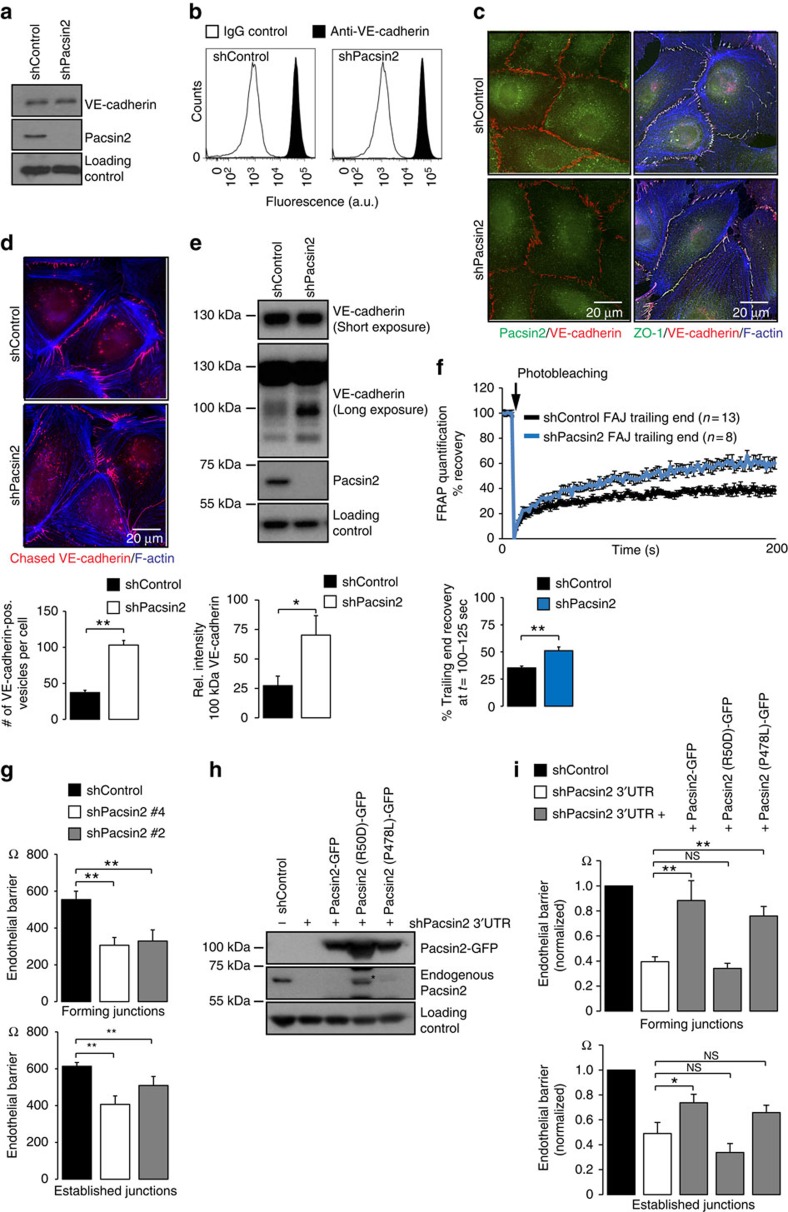
Pacsin2 controls VE-cadherin internalization and endothelial barrier. (**a**) Western blot analysis of VE-cadherin, pacsin2 and Rho GDI (loading control) in lysates of HUVECs transduced with shControl or shPacsin2#4. (**b**) Flow cytometry analysis of VE-cadherin surface expression on HUVECs transduced with shControl or shPacsin2#4. (**c**) Widefield IF images of HUVECs transduced with shControl or shPacsin2#4 stained for pacsin2 (green) and VE-cadherin (red); or ZO-1 (green), VE-cadherin (red) and F-actin (blue). See also Supplementary Fig. 2b. (**d**) Widefield IF images of HUVECs transduced with shControl or shPacsin2#4 and surface-chased VE-cadherin using monoclonal 55/7H1 and stained for F-actin (blue). Graph shows the average number±s.e.m. of VE-cadherin-positive internalized vesicles per cell. *n*≥100 cells from four independent experiments. (**e**) Western blot analysis of VE-cadherin, pacsin2 and actin (loading control) in lysates of HUVECs transduced with shControl or shPacsin2#4. Long exposure of VE-cadherin stainings show a 100 kDa fragment, which is associated with internalized VE-cadherin^46^. Graph shows the average intensity±s.e.m. of the 100 kDa VE-cadherin fragment normalized for actin loading from four independent experiments. (**f**) Line graph shows the average recovery of α-catenin-mCherry fluorescence signal as a percentage of the signal before bleaching at the trailing end of FAJs of HUVECs transduced with shControl (*n*=13 independent experiments) or shPacsin2#4 (*n*=8 independent experiments). Bar graph shows the average±s.e.m. recovered mobile fraction of α-catenin-mCherry fluorescence signal in between 100 and 125 s after bleaching. (**g**) Graph shows the average transendothelial resistance±s.e.m. of shControl, shPacsin2#4 and shPacsin2#2 transduced HUVECs during junction formation or established junctions. *n*=3 independent experiments. (**h**) Western blot analysis of pacsin2 and actin (loading control) in lysates of FACS-sorted HUVECs expressing shControl or shPacsin2 3′-UTR and rescued with GFP-tagged pacsin2, pacsin2[R50D] or pacsin2 [P478L]. Asterisk marks a degradation band of pacsin2[R50D] that runs around the same height as endogenous pacsin2. (**i**) Graph shows the average transendothelial resistance±s.e.m. of shControl, shPacsin2 3′-UTR or indicated rescued HUVECs during junction formation or established junctions. *n*=6 independent experiments. *P≤0.05; **P≤0.01 (Student's *t*-test).

To assess the role of pacsin2 in endothelial cell–cell adhesion, we next performed knockdown of pacsin2 protein expression by lentiviral expression of short hairpin RNAs (shRNAs). Two of the five tested shRNA constructs induced robust pacsin2 knockdown in HUVECs (Supplementary Fig. 2a). We detected no changes in total VE-cadherin protein levels in lysates of HUVECs depleted for pacsin2 (Fig. 9a) nor on total surface levels of VE-cadherin as determined by fluorescence-activated cell sorting (FACS; Fig. 9b). IF imaging showed that shPacsin2 knockdown cells still form VE-cadherin-based adherens junctions, including FAJs, as well as tight junctions, and we did not observe major phenotypic changes of endothelial monolayers (Fig. 9c; Supplementary Fig. 2b). To address whether pacsin2 controls VE-cadherin internalization, we first compared the fate of surface-chased VE-cadherin between shControl and shPacsin2 cells. These experiments clearly show that in the absence of pacsin2, increased levels of surface-chased VE-cadherin accumulate in internalized vesicles (Fig. 9d). Internalization of VE-cadherin is associated with the appearance of a 100 kDa fragment of VE-cadherin^46^. Western blot analysis of VE-cadherin in lysates of shControl or shPacsin2 transduced HUVECs indicate an increased quantity of the 100 kDa fragment in the absence of pacsin2 (Fig. 9e). Together these experiments evidently demonstrate that pacsin2 inhibits internalization of the VE-cadherin complex.

To investigate whether pacsin2 has a role in the polarized trafficking of α-catenin at FAJs, we next studied the mobility of α-catenin-mCherry at the trailing end of FAJs by FRAP in shControl and shPacsin2 transduced HUVECs. We find that the average mobile fraction of α-catenin-mCherry at the trailing end of FAJs is significantly higher in shPacsin2 HUVECs (51%±3.5 s.e.m.) than in shControl HUVECs (35%±1.6 s.e.m.; Fig. 9f). These experiments indicate that pacsin2 controls the polarized trafficking of the VE-cadherin complex at FAJs.

To functionally investigate the role of pacsin2 in endothelial cell–cell junctions, we analysed barrier function by measuring endothelial resistance using electric cell–substrate impedance sensing (ECIS) across endothelial cells transduced with pacsin2 or control shRNA. These experiments show that shPacsin2 cells have a significantly reduced capacity to form endothelial barriers, which is apparent both during cell–cell junction formation between recently plated cells and within established monolayers (4 and 16 h after seeding, respectively; Fig. 9g). To investigate which domains of pacsin2 are important for its barrier protective function, we knocked down pacsin2 in HUVECs expression by means of an shRNA targeting the 3′-untranslated region (UTR) of the endogenous pacsin2 messenger RNA (the shPacsin2 3′-UTR plasmid also contains a TagRFP complementary DNA). In parallel, we re-expressed GFP-tagged pacsin2, pacsin2[R50D] or pacsin2[P478L]. After transduction and puromycin selection, cells were sorted by FACS for dual GFP and TagRFP positivity. Western blot analysis shows efficient knockdown of endogenous pacsin2 and expression of the rescue constructs (Fig. 9h). Next, we investigated the capacity of these cell types to form an endothelial barrier using ECIS. These experiments confirm that pacsin2 is needed for efficient junction formation. Rescuing the shPacsin2 3′-UTR expressing cells with pacsin2-GFP significantly restores the barrier forming capacity, whereas expressing the pacsin2[R50D] mutant, which fails to concentrate at FAJs (Fig. 4a), does not mediate this effect (Fig. 9i). Expression of the pacsin2[P478L] mutant, which does become recruited to FAJs (Fig. 4a), rescues the barrier formation to a certain extent (Fig. 9i). Taken together, these data indicate that pacsin2 is required for efficient barrier formation and stabilization in an F-BAR domain dependent manner. The data point towards a protective role for F-BAR proteins during asymmetric remodelling of endothelial junctions in vascular barrier maintenance.

## Discussion

**Figure 10:**
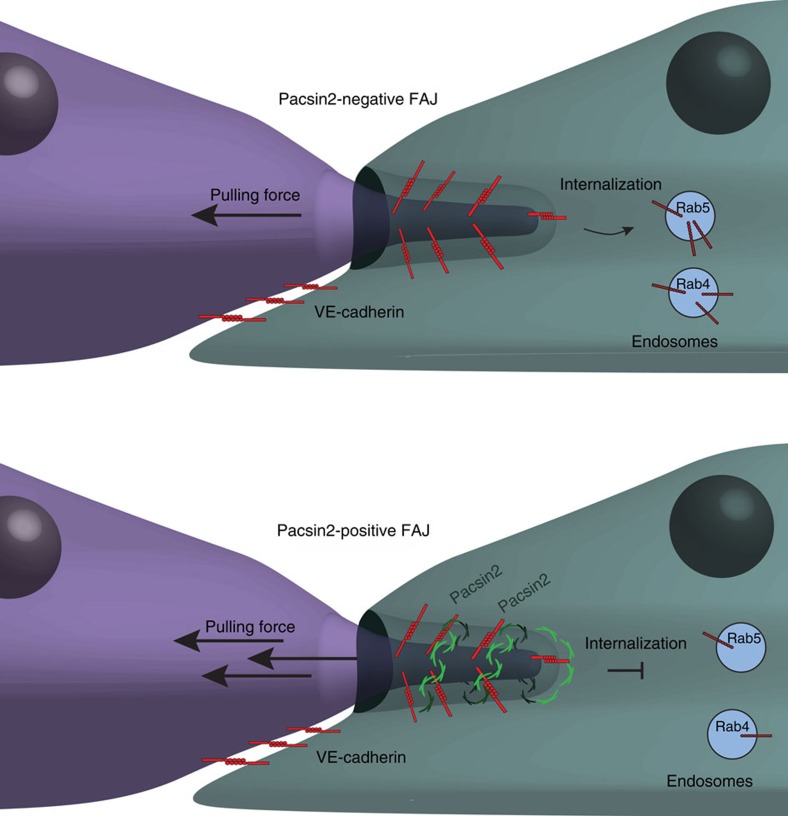
Model of pacsin2-mediated regulation of VE-cadherin at FAJs. This diagram represents our current hypothesis of how asymmetric pacsin2 recruitment regulates the trafficking of the VE-cadherin at FAJs. Triggered by unbalanced actomyosin pulling forces, causing membrane curvature at the junction, pacsin2 dimers decorate around the trailing ends of fast-moving FAJs. Pacsin2 functions to inhibit VE-cadherin internalization towards Rab5-positive and Rab4-positive endosomes.

Many recent studies underscore the importance of the activity and organization of the actomyosin cytoskeleton for endothelial cell–cell junction dynamics (reviewed in ref. 47). The regulation of junctions is crucial for development and immune surveillance, and its involvement in vascular disease is unmistakable. This study provides evidence for a novel mechanism of protection against asymmetric internalization of the VE-cadherin complex that follows unbalanced force-induced junction remodelling and is important for endothelial barrier function. Our data show that upon imbalanced pulling activity between cells (that is, when one cell pulls stronger than the other), the cell–cell junctions adopt an asymmetric geometry with a junction front where new VE-cadherin complex adhesions are formed, and a trailing end from which the VE-cadherin complex is internalized. We demonstrate that pacsin2, which is recruited to the trailing ends of a subset of FAJs, is important to maintain cell–cell adhesion during their rapid asymmetric remodelling (Fig. 10).

We identify the F-BAR protein pacsin2 as a novel regulator of endothelial cell–cell adhesion. Its recruitment to, and function at, FAJs involves its specific ability to recognize convex membrane curvature^40^. A potential alternative mechanism for its recruitment to junctions is via specific conformations of the F-actin cytoskeleton that is distinctive at FAJs^48^, and might enable a direct interaction of F-actin with the pacsin2 F-BAR domain^31^. Our super-resolution studies revealed that pacsin2 is covering around the trailing ends of FAJs and is not precisely colocalizing with the F-actin-binding proteins α-catenin and vinculin, which favors a model in which plasma membrane curves recruit pacsin2 to FAJs.

We find that pacsin2 is recruited to a subset of polarized junctions, where internalization takes place. The comparison of VE-cadherin internalization in shControl and shPacsin2 transduced cells clearly show that once pacsin2 is functionally perturbed, there are increased levels of overall internalized VE-cadherin. This increase in internalization of VE-cadherin is associated with a decreased endothelial barrier capacity, which corresponds with the notion that VE-cadherin-based adherens junctions are crucial for endothelial barrier function. The FRAP experiments comparing pacsin2-positive and pacsin2-negative FAJs indicate that once recruited to the trailing ends of FAJs, pacsin2 locally stabilizes α-catenin. This may explain the protective role of pacsin2 during junction internalization and endothelial barrier function. Others previously reported that the presence of pacsin2 at the plasma membrane inversely correlates with the recruitment of dynamin 2, a protein that mediates membrane scission^49^. Possibly, a similar competing mechanism with dynamin is responsible for the inhibitory role pacsin2 has during VE-cadherin internalization from FAJs.

Our data shows that pacsin2 is not essential for the polarized movement of VE-cadherin molecules along FAJs or their internalization. This is visible, for example, in Supplementary Movie 11, which captured the internalization process from FAJs. Pacsin2 is not continuously covering the FAJ and internalization from that FAJ is promoted once pacsin2 dissociates from the junction trailing end. In fact, in cells where pacsin2 is functionally perturbed by shRNAs, VE-cadherin internalization is strongly enhanced. Importantly, when we observe junctional recruitment of pacsin2 we consistently find it at the trailing end, where the internalization of VE-cadherin molecules occurs, and not at the FAJ front where new cell–cell adhesion complexes are being formed. Because we find a correlation between increased pacsin2 recruitment during the moments of faster polarized FAJ movements, we speculate that the pacsin2 negative, but polarized junctions, might have been pacsin2 positive earlier in their lifetime. Taken together, the data suggest that pacsin2 inhibits VE-cadherin internalization from those faster moving FAJs.

Because pacsin2 is ubiquitously expressed^30^,^34^, and we detect it also at E-cadherin- and N-cadherin-based-FAJs, we surmise that pacsin2 serves similar functions in other cell types that form classical cadherin-based cell–cell junctions. Recently, in *Caenorhabditis elegans*, the F-BAR protein SRGP-1 (an srGAP orthologue) was shown to regulate membrane dynamics during cell–cell junction formation, and its function is needed for efficient cadherin-based cell–cell adhesion^50^. Two other F-BAR proteins have been implicated in regulating cell–cell adhesion in *Drosophila*; the F-BAR proteins CIP4 and nostrin also appear to function as cadherin internalization suppressors^51^, which resembles the protective function of pacsin2 on internalization of the VE-cadherin complex. Of note, it is not yet clear whether these other F-BAR proteins are specifically recruited to certain cell–cell junction conformations, or whether they are controlled by actomyosin activity in these model systems. In epithelial cell types, depletion of junctional CIP4 impaired E-cadherin trafficking, and inhibited EGF-induced cell scattering and invasion of epithelial cancer cells^52^. Moreover, increased CIP4 protein expression levels were associated with the increased tensile adherens junctions and invasiveness of human breast cancer^52^. Together with our experiments, these data point towards tailored mechanisms regulating endothelial and epithelial cell–cell junctions by F-BAR proteins. Intriguingly, we find that nostrin is present at almost all endothelial FAJs, whereas pacsin2 is present at a subset of FAJs. There could be various reasons for this difference: there might be distinct expression levels of nostrin and pacsin2, the presence of pacsin2 at FAJs might be more transient than nostrin's or another possibility is that the degree of curvature of the F-BAR dimers is slightly different and there are more FAJs that fit the structure of nostrin. Even so, both F-BAR proteins are preferentially recruited to FAJs, and not to linear adherens junctions, which suggests that the membrane at FAJs adopts specific conformations that are recognized by these F-BAR proteins.

One of the key events implicated in inflammation, as well as in mechanical-induced remodelling of cell–cell junctions, is the trafficking of the VE-cadherin complex. Internalization of VE-cadherin occurs in response to permeability factors (for example, vascular endothelial growth factor and bradykinin), actomyosin contraction, changes in (hemodynamic) forces or transendothelial migration of immune cells^43^,^53^,^54^,^55^. Because we find that in the absence of pacsin2 the overall internalization of the VE-cadherin complex is increased, and endothelial barrier function is perturbed, we propose that pacsin2 recruitment is part of a signalling pathway that protects junctions during asymmetric dynamic events. Tensile adherens junctions are essential structures that orchestrate collective behaviour of cells in a monolayer^56^, and polarized internalization and recycling of cadherin-based adhesions underlies this phenomenon^9^. Polarized trafficking at endothelial junctions likely feeds into pathways that determine the ability of endothelial cells to adapt to the rapid changes in adhesion, pulling forces and repulsive cues^57^. Although, we observe that VE-cadherin molecules internalized from FAJs are ending up in Rab4-positive vesicles, it remains to be addressed whether pacsin2 has also a direct role in trafficking towards recycling compartments that functionally take part in newly forming cell–cell adhesions.

## Methods

### Cells and tissue

Pooled HUVECs (cultured up to passage six) from different donors (purchased from Lonza), immortalized HMEC-1 (described in ref. 58) and human BMEC-28 (described in ref. 35) were cultured in EBM-2 culture medium supplemented with EGM-2 BulletKit (Lonza) on gelatin-coated tissue flasks. HEK293T, HeLa and epithelial DU145 cells (purchased from American Type Culture Collection (ATCC)) were cultured in Iscove's modified Dulbecco's medium with L-glutamine (Lonza) supplemented with 10% fetal calf serum and antibiotics. Bone marrow-derived human mesenchymal stromal cells, a kind gift from Dr Carlijn Voermans, were isolated and cultured as described previously^59^. Human blood vessels were isolated from tissue that remains after pathology analysis of mesentery from patients that underwent intestinal tumour resection in the Academic Medical Center (Dr Mat Daemen, Amsterdam, The Netherlands) as described previously^25^. All vessels were obtained with informed consent and according to the Dutch guidelines for secondary used patient material.

### DNA constructs and lentiviral transductions

For lentiviral transductions, human pacsin2 fused at its C terminus to a GFP tag was amplified by PCR from a pcDNA6.2/C-EmGFP-DEST-pacsin2 vector (provided by Dr Ronen Zaidel-Bar) and cloned into a self-inactivating lentiviral pLV-CMV-ires-puro vector between the SnaBI and NheI restriction sites to generate pLV-CMV-pacsin2-GFP, and the pLV-CMV-pacsin2-Δ306 variant that expresses only its F-BAR domain: amino acids 1–306. The pLV-CMV-pacsin2-GFP construct was modified by replacing GFP with mCherry DNA sequence to generate pLV-CMV-pacsin2-mCherry. An arginine (CGC) to aspartic acid (GAC) mutation at amino acid position 50 in the pacsin2 F-BAR domain was generated by site-directed mutagenesis to generate pLV-pacsin2-[R50D]-GFP. A membrane localization mutant was generated by mutating methionine at position 124 and 125 (ATG ATG) to glutamic acid (GAG GAG). An SH3 mutant was generated by mutating proline at position 478 (CCG) to leucine (CTG) in pacsin2. Mouse α-catenin-mCherry and human Rab4a-GFP were cloned into the pLV-CMV-ires-puro vector using NdeI and NheI restriction sites. The lentiviral expression constructs pLV-α-catenin-Dendra2, pLV-VE-cadherin-GFP and pLV-p120-catenin-mCherry have been previously described^24^. Mammalian expression vectors for myc-tagged pacsin1, and pacsin3- and mCherry-tagged Rac1 wild type and Q61L, were previously reported^33^. Transient transfection of these constructs was performed with *Trans*IT-LT1 (Mirus) according to the manufacturers' standard protocol. Pacsin2 expression was knocked down using shRNA expression plasmids from The RNAi Consortium (TRC) library^60^. shRNA encoding pLKO.1 lentiviral vectors targeting human pacsin2 were MISSION TRC1 clones 0000037979, 0000037980, 0000037981, 0000037982 and 0000037983 from Sigma-Aldrich. The 0000037980 and 0000037983 constructs induced most efficient knockdowns in HUVECs. Non-targeting shRNA (SHC002; Sigma-Aldrich) was used as a negative control. For rescue experiments a short hairpin directed against the 3′-UTR of human pacsin2, shPacsin2-TagRFP, was designed. The shRNA was created by allowing two oligo's containing the shRNA sequence (5′-CCGGTGTCATGTCTCAGTGTCTATCTCTCGAGAGATAGACACTGAGACATGACTTTTTG-3′) to self-ligate and subsequently insert the shRNA into a modified version of the pLKO.1 backbone between the AgeI and EcoRI restriction sites. Lentiviral particles were isolated from the supernatant of HEK293T cells transiently transfected with third-generation packaging constructs and the lentiviral expression vectors. HUVECs, cultured to 80% confluency, were infected with supernatant containing lentiviral particles overnight. Cells were analysed at least 48 h after transduction. To obtain cells expressing both shPacsin2-TagRFP and pacsin2 rescue constructs, cells were sorted on FACSARIATM III cell sorter (BD) for TagRFP and GFP dual positivity.

### Antibodies and reagents

Mouse monoclonal anti-actin (clone AC-40, Cat # A3853, diluted 1/3,000 for western) anti-vinculin antibody hVIN-1 (Cat # V9131, diluted 1/100 for immunofluorescence (IF)), rabbit anti-α-catenin serum (Cat # C2081, diluted 1/400 for IF) and anti-β-catenin serum (Cat # C2206, diluted 1/400 for IF) were obtained from Sigma-Aldrich. Mouse monoclonal anti-p120-catenin antibody (clone 98/pp120, Cat # 610133, diluted 1/100 for IF), anti-β-catenin (clone 14, Cat # 610154, diluted 1/100 for IF), anti-N-cadherin (clone 32, Cat # 610920, diluted 1/100 for IF) and anti-Rho GDI (Cat # 610255, diluted 1/5,000 for western) were from BD Bioscience. Rabbit polyclonal anti-pacsin2 antibody was from Abcam (Cat # ab118330, diluted 1/100 for IF and 1/1,000 for western), and anti-pacsin1 and anti-pacsin3 rabbit sera were a kind gift from Dr Markus Ploman. Rabbit anti-nostrin was a generous gift of Dr Stefanie Oess. Monoclonal antibodies against serine 19 phosphorylated myosin light chain 2 (Cat # 3675, diluted 1/50 for IF) and Rab5 (clone C8B1, Cat # 3547, diluted 1/100 for IF) were from Cell Signaling. To detect VE-cadherin, we used the following mouse monoclonal anti-VE-cadherin antibodies: clone 75 (BD Bioscience, Cat # 610252, diluted 1/100 for IF and 1/1,000 for western), clone F-8 (Santa Cruz, Cat # SC-9989, diluted 1/100 for IF) and clone 55-7H1 directly coupled to Alexa-Fluor 647 (BD Pharmingen, Cat # 561567, diluted 1/500 for cell surface labellings); rabbit polyclonal antibody (Cayman, Cat # 160840, diluted 1/500 for IF and 1/2,500 for western) and goat polyclonal antibody (C-19 Santa Cruz, Cat # SC-6458, diluted 1/200 for IF and 1/3,000 for western). We used Acti-stain 555 (Cytoskeleton, Inc. Cat # PHDH1, diluted 1/500 for IF) or Promofluor 415-coupled phalloidin (Promokine, Cat # PK-PF415-7-01, diluted 1/250 for IF) to visualize F-actin. Secondary antibodies coupled to Alexa-Fluor 488, 568, 594 and 647 were obtained from Molecular Probes (Life Technologies) and all diluted 1/500. Bovine plasma-derived fibronectin was purchased from Sigma-Aldrich.

### IF microscopy

For standard IF staining, cells were cultured on coverslips coated with 5 μg ml^−1^ fibronectin and subsequently fixed in 4% paraformaldehyde in PBS (supplemented with 1 mM CaCl_2_ and 0.5 mM MgCl_2_; PBS^++^) for 10 min, permeabilized with 0.5% Triton X100 in PBS for 5 min and blocked for 30 min with 2% bovine serum albumin (BSA) in PBS. For the scratch assays HUVECs were cultured overnight. A scratch was made across the cell layer using a sterile pipette tip, and cells were allowed to recover and migrate for 4 h before fixation and IF staining. Primary and secondary antibody stainings were performed in 0.5% BSA in PBS for 1 h, and coverslips were mounted in Mowiol4-88/DABCO solution. For the IF stainings in Figs 7b,c and 8b,c,d,f, surface VE-cadherin was pre-labelled with an Alexa-Fluor-647-conjugated non-blocking anti-extracellular VE-cadherin antibody (mouse monoclonal 55/7H1) in EGM-2 medium at 37 °C for 30 min. After 1–2 h culturing these cells were fixed in paraformaldehyde and stained for surface VE-cadherin with mouse monoclonal 75 antibody (Fig. 7b,c) or indicated endogenous proteins. For the IF stainings in Fig. 9d, HUVECs were pre-labelled with 55/7H1 antibody in EGM-2 medium at 4 °C for 30 min and internalization was followed after 2 h of culturing the cells at 37 °C. To visualize intracellular proteins, VE-cadherin-labelled cells were permeabilized in 0.5% Triton X100 in PBS for 5 min and stained according to the standard IF protocol. Mesenteric arteries were stored in PBS^++^ at 4 °C for no longer than 24 h after surgery until further preparation as described previously^25^. IF stained samples were imaged using inverted Zeiss widefield microscopes Observer.Z1 equipped with a 63 × 1.40 Plan Apochromat oil objective, and Axiovert 200 equipped with a 63 × 1.25 numerical aperture (NA) EC Plan Neofluar oil objective, and AxioCAMMR3 and Hamamatsu Orca-R2 digital cameras. Confocal imaging of VE-cadherin and pacsin2 in HUVEC (Supplementary Movie 1), and human mesenteric arteries (Fig. 1f) were made by confocal laser scanning microscopy (Zeiss LSM 510) using a 63 × /1.4 NA oil objective and argon 488 and DPSS 561 nm lasers. For the imaging of VE-cadherin, pacsin2, Rac1 and F-actin in Fig. 3c, a LEICA TCS SP8 confocal microscope system equipped with a 63 × 1.4 NA oil objective, and 405 nm diode, 488 nm argon, 594 nm HeNe and 633 nm HeNe lasers, was used. Images were enhanced for display with an unsharp mask filter and adjusted for brightness/contrast in ImageJ (National Insitutes of Health).

### Live cell fluorescence microscopy

For live microscopy, cells were plated on Lab-Tek-chambered 1.0 borosilicated coverglass slides coated with 5 μg ml^−1^ fibronectin and imaged within microscope incubators at 37 °C and 5% CO_2_. Widefield imaging (Figs 3d, 4b and 8a; Supplementary Movies 2–8,11 and 12) was performed on an inverted Zeiss widefield Observer.Z1 microscope equipped with a 63 × 1.40 Plan Apochromat oil objective, definite focus system, and Hamamatsu Orca-R2 digital camera. For the Dendra2 photoswitching experiments (Fig. 6a; Supplementary Movie 9), a LEICA TCS SP8 confocal microscope system equipped with a 63 × 1.4 NA oil objective, and 405 nm diode, 488 nm argon and 594 nm HeNe lasers were used. Regions of interest were photoswitched by a focused 405 nm diode laser beam illumination using the LEICA FRAP module. FRAP experiments (Figs 6b and 9f; Supplementary Movie 10) were performed on a Zeiss LSM 510 Meta confocal laser scanning microscope using a 40 × 1.3 NA EC Plan Neofluar oil objective, and argon 488 and DPSS 561 nm lasers. A defined area of interest (9.64 μm^2^) was photobleached by 200 iterations using the DPSS laser at 561 nm at maximal power (15 mW). Images were enhanced for display with an unsharp mask filter and adjusted for brightness/contrast in ImageJ.

### Super-resolution microscopy

STED microscopy was performed on a Leica TCS SP8 STED 3X microscope. HUVEC monolayers were stained using the following antibodies: rabbit polyclonal anti-pacsin2 (Abcam), mouse monoclonal anti-vinculin hVIN-1 (Sigma), mouse monoclonal anti-p120-catenin clone 98/pp120 (BD Bioscience), rabbit anti-α-catenin and anti-β-catenin serum (Sigma) combined with anti-VE-cadherin mouse monoclonal F-8 (Santa Cruz) or rabbit polyclonal (Cayman) antibodies. Secondary antibodies used were STAR 580 and STAR 635P (Abberior). Samples were irradiated with a pulsed white light laser at wavelengths 587 and 633 nm. A pulsed STED laser line at a wavelength of 775 nm was used for the depletion of the 587- and 633-nm fluorophore, reaching a lateral resolution of ∼45 nm to distinguish individual fluorescent signals in these IF stainings. The signal was detected using a gated hybrid detector in counting mode. STED images were acquired using a dedicated 100 × 1.4 NA oil objective. Finally, deconvolution was performed with Huygens Professional (Scientific Volume Imaging).

### Western blot analysis

Cell lysates were prepared in reduced sample buffer and analysed by standard western blotting and enhanced chemiluminescence detection. Uncropped scans of blots are presented in Supplementary Fig. 3.

### Flow cytometry

For flow cytometry analysis in Fig. 9b, cells were collected with Accutase cell detachment solution (GE healthcare), blocked in PBS containing 1% BSA, incubated with anti-surface VE-cadherin antibody using Alexa-Fluor-647-conjugated mouse monoclonal 55/7H1 for 45 min at 4 °C, and subsequently analysed on a FACS CANTO II (Becton Dickinson).

### Electric cell–substrate impedance sensing

Endothelial barrier function was determined by measuring the electrical resistance of monolayers by ECIS. Electrode arrays (8W10E; IBIDI) were treated with 10 mM L-cysteine (Sigma) for 15 min at 37 °C and subsequently coated with 10 μg ml^−1^ fibronectin (Sigma) in 0.9% NaCl for 1 h at 37 °C. Cells were seeded and electrical resistance was continuously measured during monolayer formation at 30 kHz using ECIS model 9600 (Applied BioPhysics) and 37 °C at 5% CO_2_ incubator conditions. Resistane values were corrected for background signal that was measured in ECIS wells without cells (274 Ohm, *n*=2). In Fig. 9i, values are normalized to the resistance of shControl cells. The barrier capacity during junction formation was determined at 4 h after cell seeding, and for established monolayers at 16 h after cell seeding.

### Image and statistical analysis

For evaluations of fluorescence signals along linescans in IF images (Fig. 1a,d,g and 4a,b), fluorescence integrated intensities was measured along junctional linescans, corrected for the integrated intensity along linescans in the cytoplasm in direct proximity of the junction. A measure of 8 μm linescans were made along random junctions in the original imaging data using ImageJ. The categorization of FAJs and linear adherens junctions was based on phenotypic organization of VE-cadherin signal. FAJs are defined as interrupted and perpendicularly oriented junctions between cells. For symmetric ratio analysis at FAJs (Figs 1d and 6a), the background-corrected integrated fluorescence intensities along linescans were split into two halves of which the values of the trailing end side of junctions were divided by the values of their opposite side. A ratio of 1 represents symmetric distribution along FAJs. The evaluation of the percentage±s.e.m. of FAJs that are pacsin2- or nostrin-positive was calculated manually in widefield images (Figs 1c,g and 2). The orientation angle of pacsin2 at FAJs, in relation to the front of the scratch wound or the bottom of the image in control monolayers (Fig. 3a), was obtained by linescans from the front to the pacsin2-positive trailing end along every pacsin2-positive FAJ within a distance of four cell rows from the scratch front. To determine the average near junctional intensities of activated myosin light chain (Fig. 3b), the local integrated fluorescence instensity of p-MLC Ser19 signal was measured in fixed square areas of 18 μm^2^ at the pacsin2-positive versus pacsin2-negative side of FAJs. The values are corrected for background and normalized to maximal intensities in the overall image. To determine the speed of FAJ movement (Fig. 4b), FAJ trailing ends were tracked using the MtrackJ plugin of ImageJ in time-lapse recordings of 150 s. Pearson's split colocalization coefficients (Fig. 5b) were calculated using ImageJ. To examine colocalization of VE-cadherin with vesicles (Fig. 8g), the Mander's split coefficient of pixel intensities of VE-cadherin against vesicular proteins was determined specifically in the cytoplasm of cells and junctional regions were excluded from the analysis in background-subtracted images using automated Costes thresholding in ImageJ^61^. Quantification of the number of vesicles (Figs 8h and 9d) was performed by manually counting vesicles in the cytoplasm using the Find Maxima tool of ImageJ applying a predetermined noise tolerance value. Averages and s.e.'s of the mean were calculated and are shown in the graphs with corresponding *n* values and number of independent experiments. *P* values are the result of two-tailed, homoscedastic Student's *t*-tests.

### Data availability

The data that support the findings of this study are available from the corresponding author upon request.

## Additional information

**How to cite this article:** Dorland, Y. L. *et al*. The F-BAR protein pacsin2 inhibits asymmetric VE-cadherin internalization from tensile adherens junctions. *Nat. Commun.* 7:12210 doi: 10.1038/ncomms12210 (2016).

## Supplementary Material

Supplementary Information Supplementary Figures 1-3

Supplementary Movie 1 Pacsin2 localization at Focal Adherens Junctions. Confocal z-stack of single channel and merged IF high magnification images of FAJs in HUVECs stained for pacsin2 (green) and VE-cadherin (red). Z-stack is projected in basal-to-apical direction and each stack represents 0.227 μm in z-direction. Scale bar indicates 5 μm in xy direction. Images were acquired by confocal laser scanning microscopy (Zeiss LSM 510) using a 63x/1.4 NA oil objective.

Supplementary Movie 2 Pacsin2 dynamics during FAJ formation induced by thrombin. Time lapse recording of HUVECs expressing pacsin2-GFP (green) and α-catenin-mCherry (red). The formation of FAJs was induced by addition of thrombin at the first minute of the time lapse. Images were acquired by time-lapse widefield microscopy (Zeiss Observer Z1) using a 63x/1.4 NA oil objective. Frames were taken every 30 sec for 40 minutes.

Supplementary Movie 3 Pacsin2 dynamics during the release of cytoskeletal tension on FAJs by Y-27632. Time lapse recording of HUVECs expressing pacsin2-GFP (green) and α-catenin-mCherry (red). Tension at FAJs was reduced by addition of Rho-kinase inhibitor Y-27632 at the 90 seconds time frame of the movie. Images were acquired by time-lapse widefield microscopy (Zeiss Observer Z1) using a 63x/1.4 NA oil objective. Frames were taken every 30 sec for 20 minutes.

Supplementary Movie 4 Pacsin2 at FAJs is derived from one cell. Time lapse recording of mosaic monolayer of HUVECs expressing pacsin2-GFP (green) or pacsin2-mCherry (red). The movie shows that the FAJs contain pacsin2-GFP from the left cell, but not pacsin2-mCherry from the right cell that takes part in the FAJs. Images were acquired by time-lapse widefield microscopy (Zeiss Observer Z1) using a 63x/1.4 NA oil objective. Frames were taken every 30 sec for 18 minutes.

Supplementary Movie 5 Pacsin2 locates at the trailing ends of α-catenin at FAJs. Time lapse recording of HUVECs expressing pacsin2-GFP (green) and α-catenin-mCherry (red). The bottom cell pulls on the FAJs causing directional downwards movement of the FAJs, which is accompanied by the recruitment of pacsin2 to the trailing ends of the junctions (upper part of the junctions). Images were acquired by time-lapse widefield microscopy (Zeiss Observer Z1) using a 63x/1.4 NA oil objective. Frames were taken every 30 sec for 1 hour and 4 minutes.

Supplementary Movie 6 Pacsin2 locates at the trailing ends of VE-cadherin at FAJs. Time lapse recording of HUVECs expressing VE-cadherin-GFP (green) and pacsin2-mCherry (red). Pulling forces from the right cell cause movement of the FAJs. Pacsin2 recruitment occurs at trailing ends of the junctions (left side). Images were acquired by time-lapse widefield microscopy (Zeiss Observer Z1) using a 63x/1.4 NA oil objective. Frames were taken every 30 sec for 28 minutes.

Supplementary Movie 7 Pacsin2 locates at the trailing ends of p120-catenin at FAJs. Time lapse recording of HUVECs expressing pacsin2-GFP (green) and p120-catenin-mCherry (red). Pulling forces from the right cell cause movement of the FAJs. Pacsin2 recruitment occurs at trailing ends of the junctions (left side). Images were acquired by time-lapse widefield microscopy (Zeiss Observer Z1) using a 63x/1.4 NA oil objective. Frames were taken every 30 sec for 17 minutes.

Supplementary Movie 8 F-BAR point-mutation in pacsin2 prevents recruitment to moving FAJs. Time lapse recording of HUVECs expressing pacsin2[R50D]-GFP (green) and α-catenin-mCherry (red). The R50D point-mutation in pacsin2 prevents binding to curved membranes. The movie shows enlargements of two regions of interest (moving FAJs) from the overview image. Even 4 though clear remodeling and movement of FAJs occurs, no pacsin2[R50D] is recruited to the junctions. Images were acquired by time-lapse widefield microscopy (Zeiss Observer Z1) using a 63x/1.4 NA oil objective. Frames were taken every 30 sec for 34 minutes.

Supplementary Movie 9 Polarized dynamics of α-catenin within remodeling FAJs. Time lapse recording of HUVECs expressing α-catenin-Dendra2 shown before, and after photoswitching the front of an FAJ using a 405 confocal laser. After photoswitching a part of the FAJ, newly recruited α-catenin-Dendra2 protein (green) incorporate at the front of the FAJ, whereas α-cateninDendra2 photoswitched to red is relocated to the trailing end of the FAJ. Images were acquired by time-lapse confocal microscopy (LEICA TCS SP8) using a 63x 1.4 NA oil objective. Frames were taken every 1 minute for 12 minutes.

Supplementary Movie 10 Mobility of α-catenin differs between the front and the trailing ends of FAJs. Time lapse recording of mCherry signal in HUVECs expressing pacsin2-GFP and α-cateninmCherry. Movie shows α-catenin-mCherry signal before and after photobleaching the front of an pacsin2-positive FAJ (left movie) or the trailing end of a pacsin2-positive FAJ (right movie). In contrast to the front of FAJs, recovery of α-catenin fluorescence at the trailing end of FAJs is strongly reduced. Images were acquired by confocal laser scanning microscopy (Zeiss LSM 510) using a 63x/1.4 NA oil objective. Frames were taken every 1.57 sec for 224 sec.

Supplementary Movie 11 Pacsin2 dynamics during internalization from FAJ trailing ends. Time lapse recording of HUVECs expressing pacsin2-GFP (green) and α-catenin-mCherry (red). Movie shows the promotion of internalization from FAJs (open arrows) once recruited pacsin2 (filled arrows) dissociates from the junction trailing end. Images were acquired by time-lapse widefield microscopy (Zeiss Observer Z1) using a 63x/1.4 NA oil objective. Frames were taken every 30 sec for 32 minutes.

Supplementary Movie 12 VE-cadherin internalized from FAJs and targeted to Rab4 endosome. Time lapse recording of HUVECs expressing Rab4-GFP (green) and VE-cadherin-RFP (red). Movie shows VE-cadherin-RFP internalizing from a FAJ (followed by arrow) and being transported to Rab4-GFP-positive endosomes. Images were acquired by time-lapse widefield microscopy (Zeiss Observer Z1) using a 63x/1.4 NA oil objective. Frames were taken every 30 sec for 32 minutes.
