# Supplementary material for: The F-BAR protein pacsin2 inhibits asymmetric VE-cadherin internalization from tensile adherens junctions
Source: Nat Commun. 2016 Jul 15;7:12210. doi: 10.1038/ncomms12210 (PMC4947187; doi:10.1038/ncomms12210)
Supplement: Supplementary Information — Supplementary Figures 1-3 [file ncomms12210-s1.pdf]

SUPPLEMENTARY INFORMATION

Dorland, Malinova et al, Supplementary Figure 1

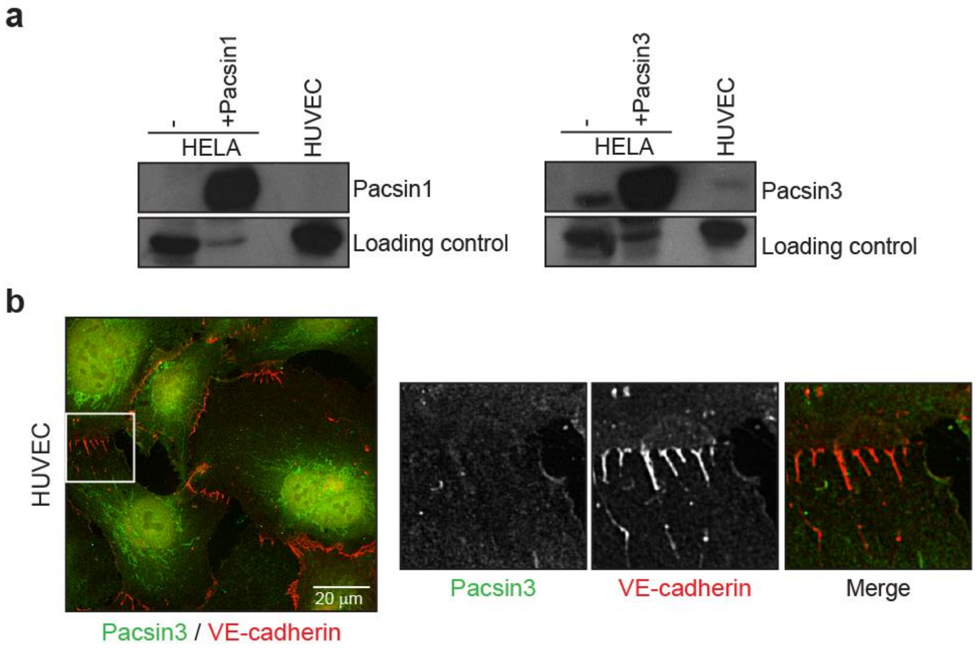

Supplementary Figure 1. (a) Western blot analysis of pacsin1, pacsin3 and actin (loading control) in HELA cells, ectopically expressing the indicated pacsin1 or pacsin3 protein, and HUVECs. **(b)** Widefield IF images of HUVECs stained for pacsin3 (green) and VE-cadherin (red).

Dorland, Malinova et al, Supplementary Figure 2

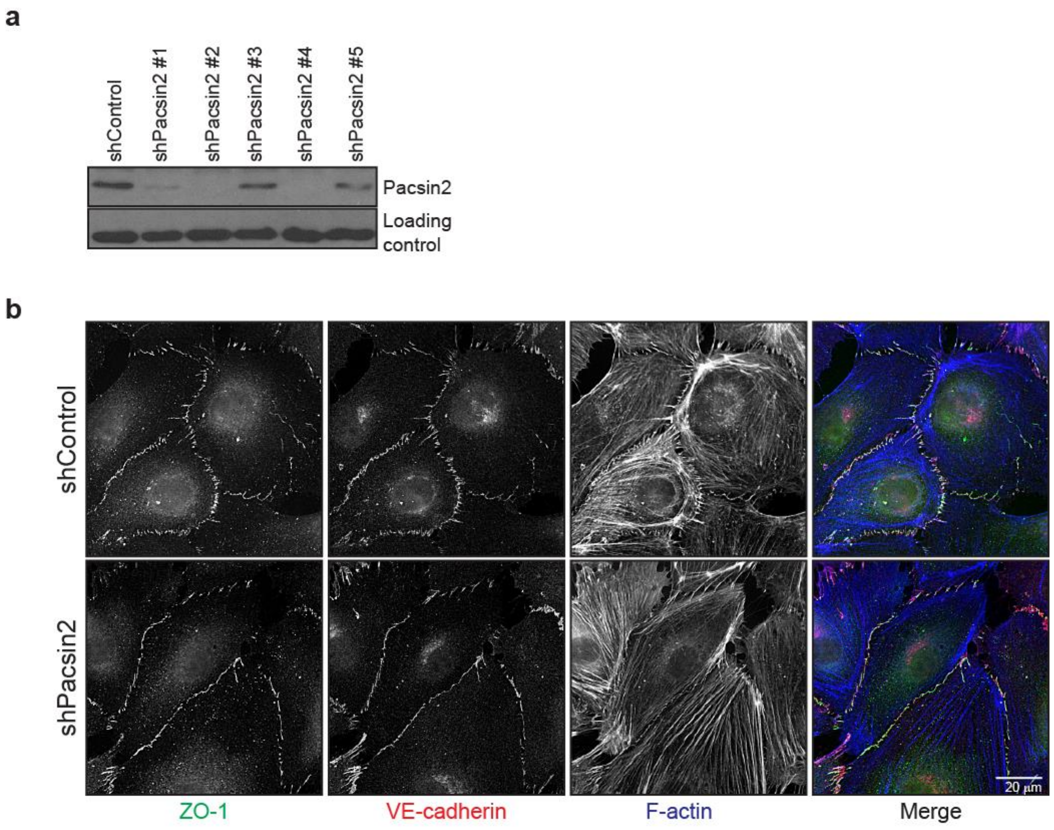

Supplementary Figure 2. (a) Western blot analysis of pacsin2 and Rho-GDI (loading control) in lysates of HUVECs lentivirally transduced with different pacsin2 shRNAs. **(b)** Widefield IF images of HUVECs transduced with shControl or shPacsin2#4 stained for ZO-1 (green), VE-cadherin (red) and F-actin (blue). This supplemental data belongs to the merged IF images of Figure 9c.

Dorland, Malinova et al, Supplementary Figure 3

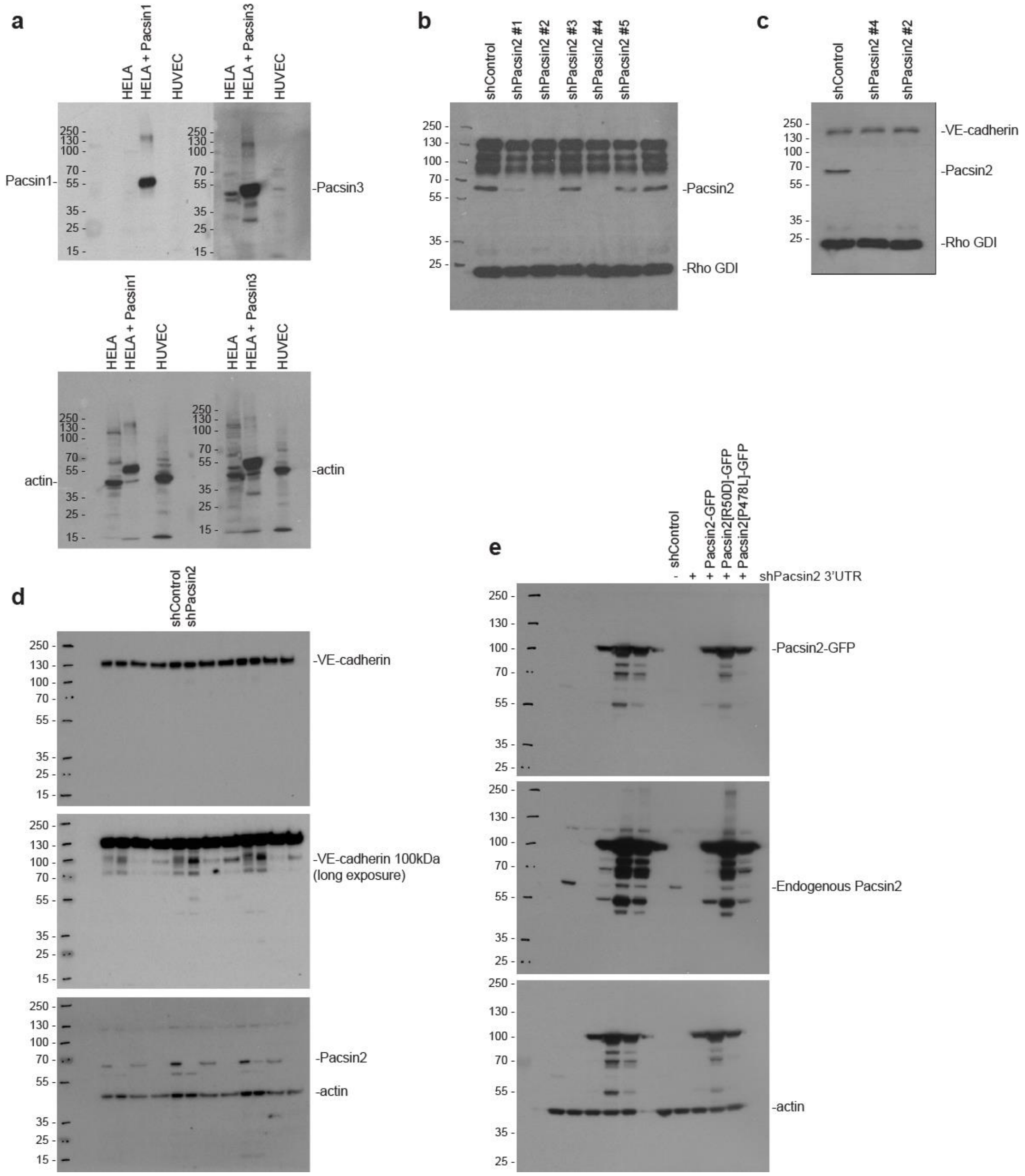

Supplementary Figure 3. Full scans of Western experiments **(a)** Supplementary Figure 1a: Western blot analysis of lysates from HELA cells, ectopically expressing the indicated pacsin1 or pacsin3 protein, and HUVECs. Blots were probed with antibodies directed against pacsin1 (upper left blot), pacsin3 (upper right blot) and actin. **(b)** Supplementary Figure 2a: Western blot analysis of lysates from HUVECs lentivirally transduced with different pacsin2 shRNAs. Lysates were prepared 96 hours after transduction. Blots were probed with antibodies directed against pacsin2 and Rho-GDI. **(c)** Figure 9a: Western blot analysis of lysates from HUVECs transduced with shControl, shPacsin2#4 or shPacsin2#2, 68 hours after transduction. Blot was probed with antibodies directed against VE-cadherin pacsin2 and Rho GDI. **(d)** Figure 9e: Western blot analysis of lysates from HUVECs transduced with shControl or shPacsin2#4, 96 hours after transduction. Blots were probed with antibodies directed against VE-cadherin, pacsin2 and actin. Normal and longer exposures are shown for the VE-cadherin probing. **(e)** Figure 9h: Western blot analysis of lysates from FACS-sorted HUVECs expressing shControl or shPacsin2 3'UTR and rescued with GFP-tagged pacsin2, pacsin2[R50D] or pacsin2[P478L]. Blots were probed with antibodies directed against pacsin2 and actin.
